# Ni(OH)_2_-Type Nanoparticles Derived from Ni Salen Polymers: Structural Design toward Functional Materials for Improved Electrocatalytic Performance

**DOI:** 10.1021/acsami.2c06147

**Published:** 2022-07-15

**Authors:** Monika Mierzejewska, Kamila Łępicka, Jakub Kalecki, Wojciech Lisowski, Piyush Sindhu Sharma

**Affiliations:** Institute of Physical Chemistry, Polish Academy of Sciences, Kasprzaka 44/52, 01-224 Warsaw, Poland

**Keywords:** electrocatalyst, ethanol, metal nanoparticle, electrodeposition, heterogeneous electrocatalyst

## Abstract

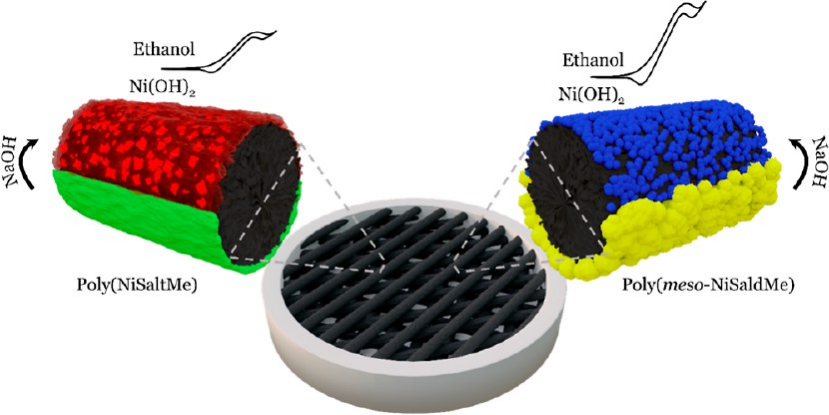

Herein, we report the potential-driven electrochemical transformation carried out in basic media of two Ni^2+^ salen polymers, (poly(NiSalen)s), abbreviated as poly(*meso*-NiSaldMe) and poly(NiSaltMe). These two polymers, with different configurations of methyl substituents on the imine bridge, were used as precursors for the preparation of electrocatalytically active nickel hydroxide [Ni(OH)_2_]-type nanoparticles (NPs) anchored in the polymeric matrix as poly[SalenNi(OH)_2_]. The use of potentiodynamic and potentiostatic electropolymerization conditions for the deposition of polymeric precursors allowed us to control the molecular architecture of poly(NiSalen)s and NPs derived from them. Thus, we obtained different arrangements of NPs embedded in morphologically different poly(Salen) matrixes, indicating their electrocatalytic activity toward ethanol to different extents. Moreover, we found a direct relationship between the electrochemical stability of the poly(NiSalen) precursors operating in the organic solvent-based electrolyte solutions and the easiness of their transformation into Ni(OH)_2_ NPs operating in the aqueous alkaline media. Poly(NiSalen)s and Ni(OH)_2_-type NPs were characterized by X-ray photoelectron spectroscopy, scanning electron microscopy, and transmission electron microscopy.

## Introduction

There is a strong emphasis on finding greener energy sources to substitute fossil fuels because of the ongoing energy crisis. Because of that, the alcohol fuel cell (AFC) is considered one of the most prominent alternatives.^1^ AFCs are compact and suitable for modern portable gadgets like mobile phones or laptops.^2^ Moreover, ethanol utilized as a fuel in AFCs is considered the green fuel that faces the fact toward CO_2_ neutrality in closed circuits. The complete combustion of ethanol emits only CO_2_ and water vapor that can be consumed during plant photosynthesis. The higher energy density and lower toxicity of ethanol in comparison to methanol, its abundant sources, ease of transport, and storage direct its application as a cheap and standard fuel in AFCs.^3^

However, such fuel cells usually involve noble metals as electrocatalytically active materials, for instance, Pt.^4^ Unfortunately, Pt-based catalysts suffer from the poisoning effect caused by adsorbed side products of electrocatalytic reactions.^5,6^ Ultimately, the high price of Pt-, Au-, and Pd-based catalysts motivated further development of AFCs devoted to using less precious metal alloys together with a smaller amount of noble metals to make the system more cost-effective.^7−9^ Furthermore, attempts were made to deal with the limitation of Pt abundance by replacing Pd or Ru.^10,11^ However, this approach proposed only the replacement of one precious metal with another.^10^

The electrochemical reactivity of Ni^2+^ species in aqueous alkaline media was utilized for various electrochemical applications,^12^ including electrocatalytic electro-oxidation of small organic molecules, for example, for energy conversion,^13^ electrochemical sensing,^14^ and energy storage.^15,16^ The molecular structures of electrochemically prepared Ni^2+^ hydroxide [Ni(OH)_2_]-based electrodes and their comparison with chemically prepared ones have been interesting for many years.^17−19^ The electrochemical oxidation of Ni^2+^ hydroxide to Ni^3+^ oxy-hydroxide and the subsequent reduction back to Ni^2+^ hydroxide^20^ were assigned as the products of Ni^2+/^Ni^3+^ electrochemical switching in alkaline media.^21,22^ Moreover, it was found that the crystal microstructure of Ni^2+^ hydroxide^17,20^-based electrodes determined their electron conductivity limitations under electrochemically driven Ni^2+/^Ni^3+^ redox reactions.^23^ Thus, this prompted scientific efforts to prepare differently structured Ni(OH)_2_-type electrodes with increased catalytic activity at a lower cost. Hence, a few structurally different Ni(OH)_2_-type materials derived from Ni^2+^ complexes were investigated to facilitate the electrocatalytic oxidation of alcohols in alkaline media.^24−27^ Most of these studies attempted to link the electrochemical and electrocatalytic performances of Ni(OH)_2_-type electrodes derived from various Ni^2+^ organic complexes.^24−27^ However, the relations between the newly introduced chemical structure changes of Ni^2+^ complexes used to fabricate catalysts indicating improved properties were not established.

The highest possible electrochemically active surface area (*A*_ECSA_) of a catalyst is its unambiguously desired property that can be achieved by preparing a functional material featuring a uniform distribution of nanoparticles (NPs). Moreover, the appropriate control of the molecular structure and thus the interactions between NPs and the surroundings, for example, functional groups attached, can ensure control over the electrocatalyst durability and its spatial structure.^28^

The group of poly(NiSalen)s is assigned to polymer semiconductors that reveal a mixed redox and π-conjugated conductivity in a moderately electron-donating medium.^29,30^ Their Faradaic charge conduction mechanism can be explained in a simplified way as the transport of delocalized valence electrons within the model of a Peierls distorted polymer lattice, such as that for polyphenylene-type polymers.^31^ Importantly, this charge transport occurs only in a particular continuous range of potentials involving oxidized polymer forms (bisphenolic radicals and bisphenolic cations), revealing the p-type of electrochemical doping,^32^ where no metal-centered oxidation, such as Ni^2+^/Ni^3+^, is observed.^33−35^ In a moderately electron-donating medium, poly(NiSalen) behaves like a polyphenylene, with the Ni^2+^ ion acting as a bridge between biphenylene moieties.^36^

The electrochemical transformation of electrochemically deposited poly(NiSalen) in aqueous alkaline electrolytes was recently adopted as a new method for preparing Ni^2+^-based Ni(OH)_2_-type NP electrocatalysts.^37,38^ In such cases, hydrolysis of poly(NiSalen)s resulted in the formation of electrode coatings consisting of uniformly distributed Ni(OH)_2_ NPs. However, the reusability of such NPs was limited because of the poisoning effect caused by the product.^37^ Moreover, the effect of the molecular and chemical structure tuning of Ni^2+^ salen monomers and their polymers toward its catalytic activity after NP generation was not considered. Therefore, further study is required to achieve better performing electrocatalytic materials.

However, there are challenges in designing stable poly(NiSalen)s related to (i) their chemical structure tuning and (ii) tailoring their molecular architecture to control properties for dedicated application, for instance, for the preparation of precursors for electrocatalytically active Ni(OH)_2_-type NPs, functional electrochromic materials,^35,39^ or capacitor electrodes.^30,40^ The possibility of electrodeposition condition tuning, for example, changing the scan rate, number of cycles, and the potential range of electropolymerization, enables the change in properties of poly(NiSalen)s to prepare electroactive films with a consistent molecular architecture.^41^

One of the most widely used methods of preparation of polymer films on conducting substrates is electropolymerization under potentiodynamic (PD) or potentiostatic (PS) conditions.^42,43^ Previously, the electrochemical stabilities of potentiodynamically electrodeposited poly(*meso*-NiSaldMe) and poly(NiSalen) films were compared in the moderately electron-donating organic solvent-based supporting electrolyte solution.^30^ The high electrochemical stability of poly(*meso*-NiSaldMe) was studied in detail by the electrochemical quartz crystal microbalance method and electrochemical PeakForce quantitative nanomechanical mapping AFM.^41^

Herein, poly(NiSaltMe)^33,34^ and poly(*meso*-NiSaldMe)^35^ with electrochemically tuned morphologies were applied for the first time as a precursor for the preparation of Ni(OH)_2_-type NP-based electrocatalysts for ethanol electro-oxidation. The potential-driven electrochemical transformations of poly(*meso*-NiSaldMe) and poly(NiSaltMe) films into Ni(OH)_2_-type NPs carried out in basic media were extensively studied. The time needed for the complete transformation of poly(NiSalen) films into Ni(OH)_2_-type NPs correlated well with their electrochemical stability in the moderate electron-donating organic solvent-based supporting electrolyte solution. A relationship between the structure–reactivity requirements toward the electrochemical stability of the poly(NiSalen) precursors and NPs derived from them operating in the basic media was defined.

Poly(NiSalen) films and Ni(OH)_2_-type NPs were characterized by X-ray photoelectron spectroscopy (XPS), scanning electron microscopy (SEM), and transmission electron microscopy (TEM). Furthermore, the effect of additional reinforcement of poly(NiSalen) precursors with reduced graphene oxide (RGO) was investigated.

## Experimental Section

### Chemicals

Tetra(*n*-butyl)ammonium hexafluorophosphate, (TBA)PF_6_; ethanol, EtOH; and anhydrous acetonitrile used were of electrochemical grade and purchased from Sigma-Aldrich. Sodium hydroxide (NaOH) used was of analytical grade and purchased from POCH. The carbon paper FuelCellsETc was purchased from College Station, TX. The NiSaltMe and *meso*-NiSaldMe monomers were synthesized according to the procedure described elsewhere.^30,35,40^

### Electrochemical Cell Configuration and Electrodes

All electrochemical measurements were carried out in a three-electrode cell. Two types of carbon paper electrodes (CPEs) of 5 mm diameter were used as the working electrode, that is, bare CPE and chemically reduced GO-coated CPE (geometrical area of ∼0.196 cm^2^).^40^ The preparation steps of laminated CPE are described elsewhere.^40^ For modification of the CPE electrode, RGO dispersed in DMF was drop-coated. The chemically reduced GO was prepared by reducing it with lithium aluminum hydride.^44^ After evaporation of DMF under reduced pressure, RGO-coated CPE electrodes were rinsed with acetonitrile and stored in a dry place. The Pt mesh and silver wire were used as counter and pseudo-reference electrodes, respectively. When the electrochemical experiment was performed in aqueous conditions, the Ag/AgCl (3 M KCl) electrode from Metrohm was employed as a reference electrode.

### Characterization of Polymeric Precursors and Nanoparticles

The SP300 electrochemistry system of Bio-Logic Science Instruments, controlled using EC-Lab software from the same manufacturer, was used for electrochemical measurements.

Polymer film- and NP-coated electrodes were imaged by SEM using an FEI Nova NanoSEM 450. TEM analysis was performed using a Talos F200X microscope equipped with Super X, an energy-dispersive X-ray spectroscopy (EDS) detector. For TEM analysis, NP electrocatalysts were scratched from the electrode surface and placed on the copper grid.

XPS spectra were measured with the PHI 5000 VersaProbe Scanning ESCA Microprobe using monochromatic A1 Kα radiation (*h*ν = 1486.4 eV). The XPS data were generated with a 100 μm in diameter X-ray beam and collected from a 500 × 500 μm irradiated area. High-resolution (HR) XPS spectra were collected with a hemispherical analyzer at a pass energy of 23.5 eV, an energy step of 0.1 eV, and a photoelectron take-off angle of 45° to the surface plane. XPS data were analyzed with CasaXPS (v. 2.3.19) software. Peaks were fitted using a Shirley background and a Gaussian/Lorentzian peak shape character. The binding energy (BE) scale of all detected spectra was referenced by setting the BE of C 1s signal to 284.8 eV.

### Procedures

The polymer films of poly(NiSaltMe) and poly(*meso*-NiSaldMe) were grown from the 1 mM monomer and 0.1 M (TBA)PF_6_ acetonitrile solution on the CPEs or the CPEs drop coated with the RGO film, either under (i) multi-scan PD conditions in the potential range of 0.0–1.3 V vs Ag/Ag^+^ (on bare CPE) or 0.0–1.4 V vs Ag/Ag^+^ (on CPE drop coated with RGO) at a low scan rate of 2 mV s^–1^ and a high scan rate of 100 mV s^–1^ or under (ii) PS conditions at 1.3 V vs Ag/Ag^+^. The PS depositions were accomplished by passing similar charges as passed during PD depositions at 2 and 100 mV s^–1^.

That way, 12 different poly(NiSalen) precursor samples were prepared, as described below:

#### Poly(NiSaltMe) Precursor Films

(1a and 1a′) Potentiodynamically deposited precursor films at 100 mV s^–1^ for 29 and 16 cycles, named poly(NiSaltMe)-PD_high_ and poly(NiSaltMe)-PD_high′_, respectively.

(2a and 2a′) Potentiodynamically deposited precursor films at 2 mV s^–1^ for three and two cycles, named poly(NiSaltMe)-PD_low_ and poly(NiSaltMe)-PD_low′_, respectively.

(3a) Potentiostatically deposited precursor film, named poly(NiSaltMe)-PS_high_, with the amount of charge deposited corresponding to the polymerization charge of poly(NiSaltMe)-PD_high_.

(4a) Potentiostatically deposited precursor film, named poly(NiSaltMe)-PS_low_, with the amount of charge deposited corresponding to the polymerization charge of poly(NiSaltMe)-PD_low_.

#### Poly(*meso*-NiSaldMe) Precursor Films

(1b and 1b′) Potentiodynamically deposited precursor films at 100 mV s^–1^ for 29 and 16 cycles, named poly(*meso*-NiSaldMe)-PD_high_) and poly(*meso*-NiSaldMe)-PD_high′_, respectively.

(2b and 2b′) Potentiodynamically deposited precursor films at 2 mV s^–1^ for three and two cycles, named poly(*meso*-NiSaldMe)-PD_low_ and poly(*meso*-NiSaldMe)-PD_low′_, respectively.

(3b) Potentiostatically deposited precursor film, named poly(*meso*-NiSaldMe)-PS_high_, with the amount of charge deposited corresponding to the polymerization charge of poly(*meso*-NiSaldMe)-PD_high_.

(4b) Potentiostatically deposited precursor film, named poly(*meso*-NiSaldMe)-PS_low_, with the amount of charge deposited corresponding to the polymerization charge of poly(*meso*-NiSaldMe)-PD_low_.

The potential-driven multi-scan electrochemical transformation of all poly(NiSalen) precursor films into NPs was carried out in 0.2 M NaOH solution at a scan rate of 20 mV s^–1^ in the potential range of 0.0 to 1.20 V vs Ag/AgCl until the oxidation and reduction peaks of Ni^2+^/Ni^3+^ were no longer grooving. After NP preparation, electrodes were rinsed with Milli-Q water (18.2 Ω) and stored in dry conditions. The electrocatalytic activity studies were investigated in 0.2 M NaOH solution.

Electrochemical impedance spectroscopy (EIS) measurements were registered at different potentials corresponding to chosen electroactivity states of Ni(OH)_2_-type NPs derived from poly(NiSaltMe)-PS_high_ and poly(*meso*-NiSaldMe)-PS_low_ in (i) bare 0.2 M NaOH_aq_ and (ii) 0.2 M NaOH_aq_ containing 0.3 M ethanol. Before the EIS measurements, these NP-coated CPEs were equilibrated by applying a selected potential for 2 min. After that time, the current reached equilibrium and then EIS measurements were performed at the voltage amplitude of 10 mV in the frequency range of 10 kHz to 10 mHz. Before each EIS measurement, electrodes were CV-cycled from 0.0 to 1.0 V in 0.2 M NaOH_aq_, at the scan rate of 50 and 5 mV s^–1^. EIS spectra were analyzed by numerical randomization + Simplex data fitting to the modified Randles–Ershler equivalent circuits containing different configurations of electrical element-mimicking properties of prepared materials. EIS data were acquired and then analyzed using the Zfit function implemented in EC-Lab software, version 11.31 from Bio-Logic. The minimum value of χ^2^ (close to zero) evaluated the accuracy of equivalent circuit fitting to experimental data.

### Quantum Chemical Calculations

Geometries of the structures of the single catalytic center of Ni(OH)_2_-type NPs embedded in poly(SaltMe) and poly(*meso*-SaldMe) matrixes were optimized with semiempirical PM6-D3 (PM6 Hamiltonian),^45^ with the Grimme empirical dispersion D3^46^ method implemented in the quantum chemical software package MOPAC2016.^47^ The output geometries obtained from semiempirical calculations were used as an input for density functional theory (DFT) calculations.

DFT calculations were performed with the Tao–Perdew–Staroverov–Scuseria functional.^48^ As in the case of semiempirical calculations, Grimme empirical dispersion D3^46^ was included. As a basis-set, 6-31^++^G(d,p) (Gaussian double-zeta valence basis-set with dispersion and diffuse functions) was used.

## Results and Discussion

### Different Conditions of Polymer Film Electrodeposition

It was observed that conducting polymer films can be deposited with different morphologies by tuning electropolymerization conditions.^49^ The polymer film deposited under PD conditions at a low scan rate is usually densely packed with relatively large globular structures. In contrast, polymer film deposition at a high scan rate can result in a more uniform packing of smaller globules. Different morphologies of salen-based polymer films can affect their electrochemical stability, and, by this, the properties of NPs derived from them.

**Figure 1:**
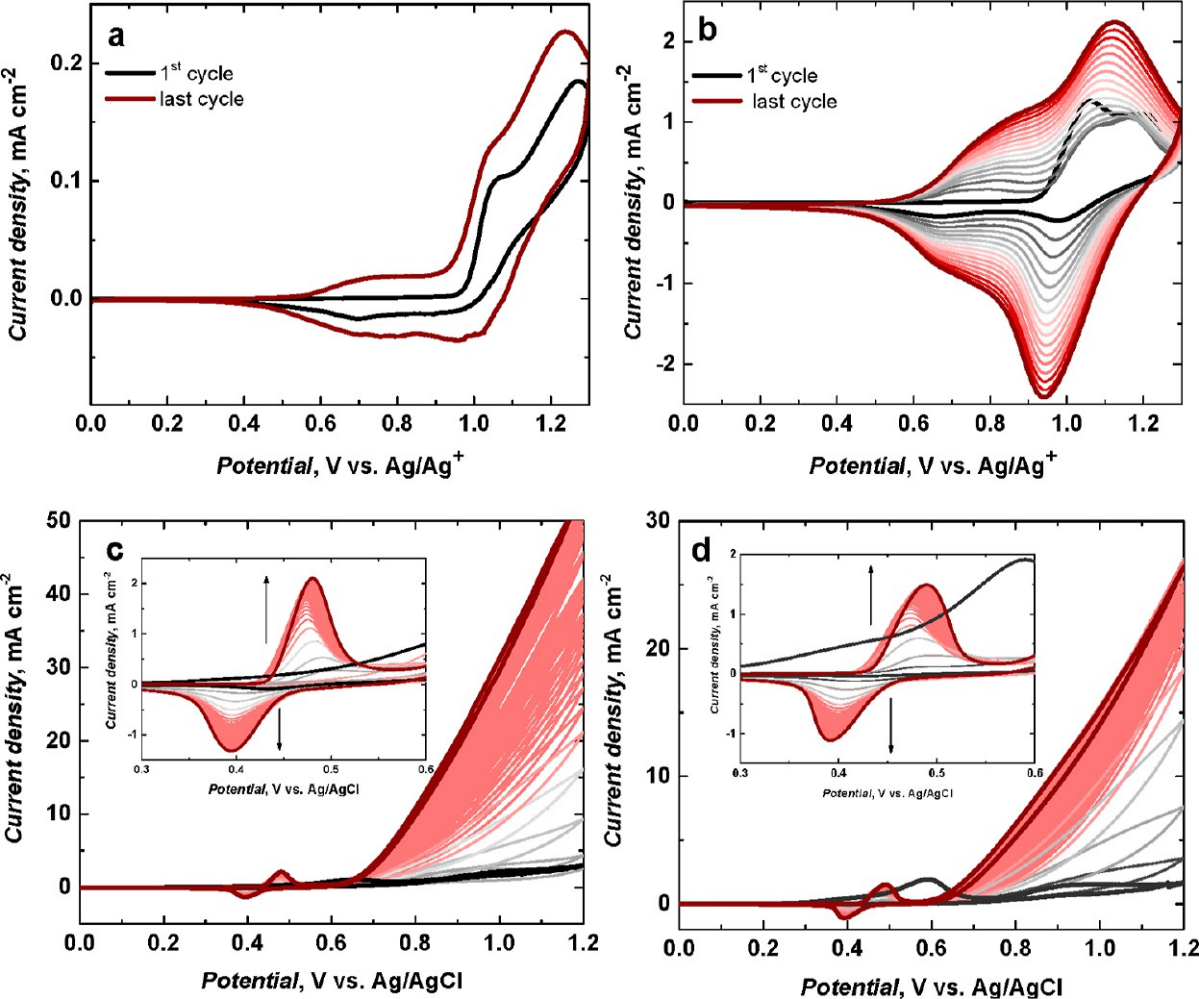
Multi-cyclic PD curves of oxidative electropolymerization of 1 mM Ni-SaltMe in an acetonitrile solution of 0.1 M (TBA)PF_6_ at (a) 2 and (b) 100 mV s^–1^. Multi-cyclic curves of potential-driven NP generation from the (c) poly(NiSaltMe)-PD_low′_ and (d) poly(NiSaltMe)-PD_high′_ films performed at 20 mV s^–1^ in 0.2 M NaOH.

Toward that, herein, poly(NiSalen) films were deposited under different electropolymerization conditions, that is, PD at low and high scan rates and PS with high and low charges passed during PD depositions. Figure 1 shows oxidative electropolymerization of NiSaltMe at a low scan rate, that is, the CPE coating with poly(NiSaltMe)-PD_low′_ (Figure 1a), and at a high scan rate, that is, the CPE coating with poly(NiSaltMe)-PD_high′_ (Figure 1b). Conducting polymer film deposition was evident by the consecutive current increase in the subsequent CV cycles. In the first cycle of low (Figure 1a) and high (Figure 1b) scan rates, PD polymerizations of NiSaltMe, two anodic and two cathodic peaks were observed. The first anodic peak centered at ∼1.00 V (Figure 1a) and ∼1.05 V (Figure 1b) corresponded to NiSaltMe monomer’s electro-oxidation on the CPE surface to bisphenolic cation radicals. The second oxidation peak was observed at ∼1.25 V (Figure 1a) and ∼1.15 V (Figure 1b). This peak was characteristic of bisphenolic cation radical oxidation to bisphenolic cations. Whereas the first cathodic peak at ∼1.00 V (Figure 1a) and ∼0.95 V (Figure 1b) and the second cathodic peak at ∼0.70 V (Figure 1a) and ∼0.65 V (Figure 1b) corresponded to the bisphenolic cation reduction to bisphenolic cation radicals and the consequent bisphenolic cation radical reduction to the neutral forms, respectively. After the first cycle of PD polymerization, the polymer film started to grow, which was evident from the appearance of broad anodic and cathodic waves growing in the subsequent cycles (characteristic of initial electrochemical doping and generation of charge transport species—bisphenolic radical cations inside the conducting polymer layer already deposited on the electrode),^32,33,35^ both centered between 0.50 and 0.85 V for poly(NiSaltMe)-PD_low′_ (Figure 1a) and poly(NiSaltMe)-PD_high′_ (Figure 1b). The charges passed during all PD polymerizations are summarized in Table 1.

**Table 1:** Efficiency of Electro-oxidation of Ethanol over Ni(OH)_2_ NPs Generated from Different Poly(Salen)s^a^

	poly(NiSaltMe)			poly(*meso*-NiSaldMe)		
electrodeposition conditions	charge (mC cm^–2^)	number of cycles for NP generation	sensitivity (mA cm^–2^ M^–1^)	charge (mC cm^–2^)	number of cycles for NP generation	sensitivity (mA cm^–2^ M^–1^)
PD 2 mV s^–1^	83.3	120	49.0	117.3	280	62
PD′ 2 mV s^–1^	51.3	55	44.3	69.3	150	60
PS	81.6	40	47.0	137.1	80	67
PD 100 mV s^–1^	181.1	130	37.0	178.5	250	54
PD′ 100 mV s^–1^	88.2	62	53.3	94.3	120	54
PS	176.4	57	73.3	188.7	145	69.4

a PS—potentiostatic deposition; PD—potentiodynamic deposition.

The poly(NiSaltMe)-PS_low_ (Figure S1a) and poly(NiSaltMe)-PS_high_ (Figure S1a′) films were prepared by electropolymerization under PS conditions. The constant potential of 1.3 V was kept for the time needed to pass the required charge, corresponding to the PD electropolymerization charge of poly(NiSaltMe)-PD_low_ and poly(NiSaltMe)-PD_high_. The two-electron oxidation of NiSaltMe and the consequent two-electron transfer employing the continuous formation of fully oxidized poly(NiSaltMe) species during PS electropolymerization were ensured by keeping the potential value of 1.3 V versus Ag/Ag^+^ for poly(NiSaltMe)-PS_low_ (Figure S1a) and poly(NiSaltMe)-PS_high_ (Figure S1a′) for 877 and 1036 s, respectively.

**Figure 2:**
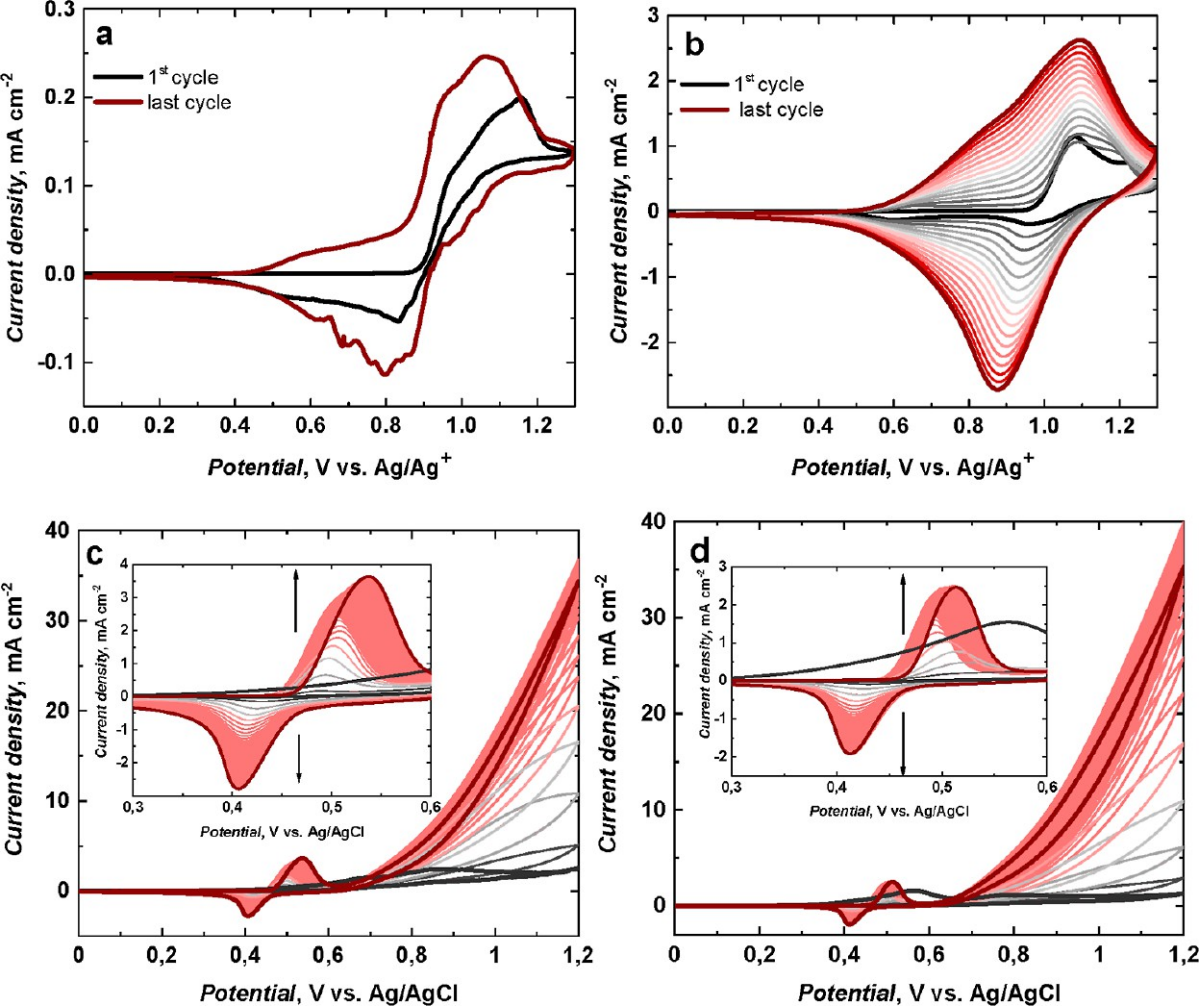
Multi-cyclic PD curves of oxidative electropolymerization of 1 mM *meso*-NiSaldMe in an acetonitrile solution of 0.1 M (TBA)PF_6_ registered at (a) 2 and (b) 100 mV s^–1^. Multi-cyclic curves of the potential-driven NP generation from the (c) poly(*meso*-NiSaldMe)-PD_low′_ and (d) poly(*meso*-NiSaldMe)-PD_high′_ films performed at 20 mV s^–1^ in 0.2 M NaOH.

Figure 2a shows the oxidative electropolymerization of *meso*-NiSaldMe at a low scan rate, that is, the CPE coating with poly(*meso*-NiSaldMe)-PD_low′_ (Figure 2a), and at a high scan rate, that is, the CPE coating with poly(*meso*-NiSaldMe)-PD_high′_ (Figure 2b). The deposition of conducting films of poly(*meso*-NiSaldMe)-PD_low′_ and poly(*meso*-NiSaldMe)-PD_high′_ was evident from the consecutive current increase in the subsequent CV cycles. Two anodic and two cathodic peaks were observed in the first cycle of PD polymerization of *meso*-NiSaldMe at a low scan rate (Figure 2a). The first anodic peak centered at ∼0.97 V (Figure 2a) corresponded to the initial electro-oxidation of *meso*-NiSaldMe on the CPE surface to the bisphenolic cation radical. The second peak observed at ∼1.15 V (Figure 2a) was characteristic of the bisphenolic cation. The first and the second cathodic peaks were centered at ∼1.00 and ∼0.80 V, respectively, corresponding to the bisphenolic cation reduction to bisphenolic cation radicals and the consequent bisphenolic cation radical reduction (Figure 2a). In the second cycle of PD polymerization of *meso*-NiSaldMe at a low scan rate (Figure 2a), the shift of two anodic and two cathodic peaks toward more negative potentials and the appearance of broad anodic and cathodic waves, both centered between 0.50 and 0.75 V, indicated the successful deposition of the poly(*meso*-NiSaldMe)-PD_low′_ film on the CPE surface.

In the first cycle of PD polymerization of *meso*-NiSaldMe at a high scan rate (Figure 2b), two anodic and two cathodic peaks were observed at ∼1.09 and ∼1.20 as well as at ∼1.19 and ∼0.98 V, respectively. Because of the fast charge transport in poly(*meso*-NiSaldMe)-PD_high′_ (Figure 2b), the broad anodic and cathodic waves and two anodic and two cathodic peaks appearing at subsequent cycles were not much pronounced as those observed in the second cycle for poly(*meso*-NiSaldMe)-PD_low′_ (Figure 2a). Moreover, the facilitated fast charge transport of poly(*meso*-NiSaldMe)-PD_high′_ in comparison with poly(NiSaltMe)-PD_high′_ was reflected in the amount of charge that passed within the same number of PD polymerization cycles (16 cycles, Table 1).

The poly(*meso*-NiSaldMe)-PS_low_ (Figure S2a) and poly(*meso*-NiSaldMe)-PS_high_ (Figure S2a′) films were prepared by electropolymerization under PS conditions. The constant potential of 1.3 V was kept for the time needed to pass the charge corresponding to the PD electropolymerization charge of poly(*meso*-NiSaldMe)-PD_low_ and poly(*meso*-NiSaldMe)-PD_high_ films. The two-electron oxidation of *meso*-NiSaldMe and the consequent two-electron transfer involving the continuous formation of oxidized species in poly(*meso*-NiSaldMe) during PS electropolymerization was ensured by keeping the constant potential value of 1.3 V vs Ag/Ag^+^ for poly(*meso*-NiSaldMe)-PS_low_ (Figure S2a) and poly(*meso*-NiSaldMe)-PS_high_ (Figure S2a′) for 782 and 2107 s, respectively.

### Ni(OH)_2_-Type NP Generation

**Scheme 1:**
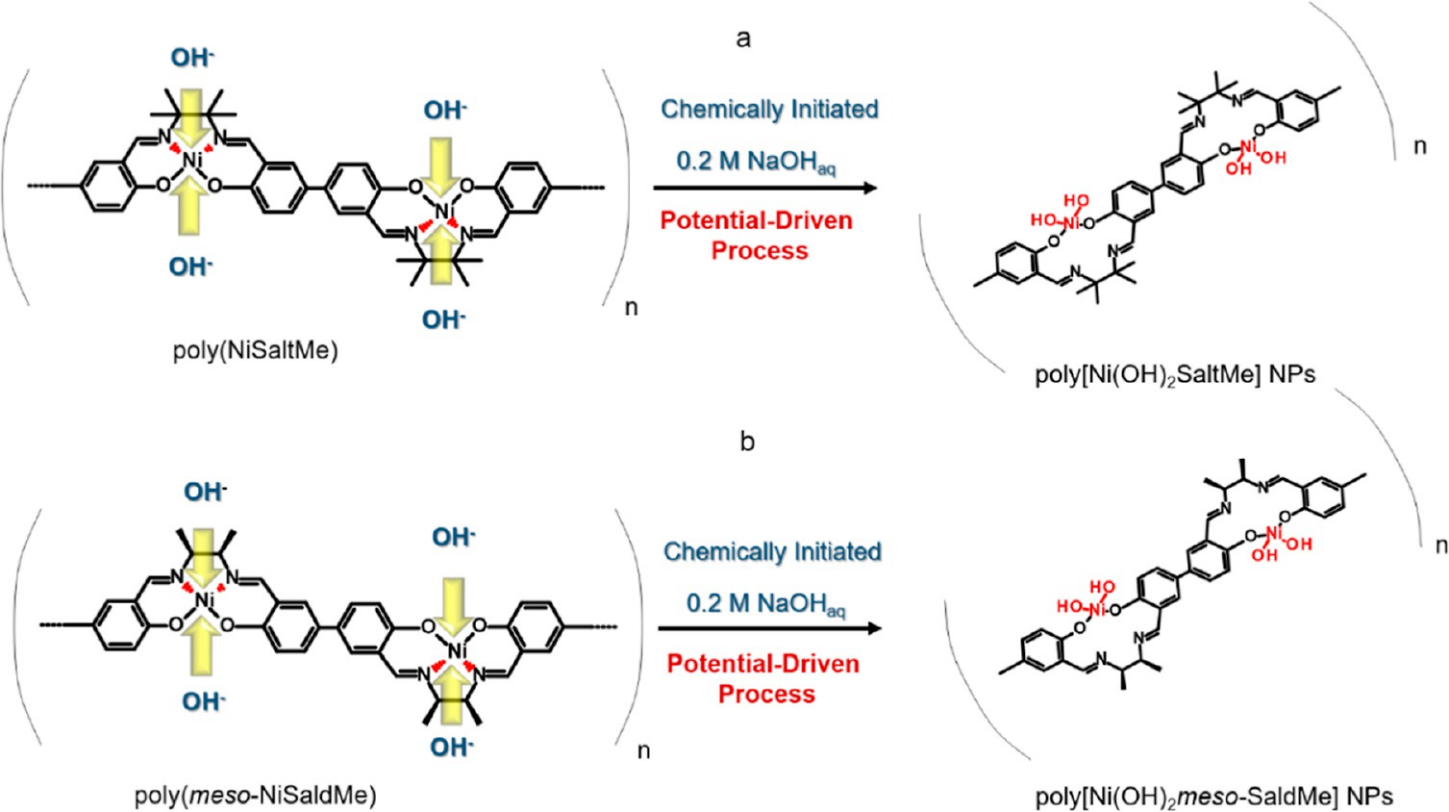
Illustration of the Electrochemically Driven Basic Hydrolysis Process Showing the Breaking of Square Planar Geometry of Ni^2+^ in (a) Poly(NiSaltMe) and (b) Poly(*meso*-NiSaldMe)

Ni(OH)_2_-type NPs were prepared by the potential-driven electrochemical transformation of poly(NiSaltMe)-PD_low′_ (Figure 1c), poly(NiSaltMe)-PD_high′_ (Figure 1d), poly(*meso*-NiSaldMe)-PD_low′_ (Figure 2c), poly(*meso*-NiSaldMe)-PD_high′_ (Figure 2d), poly(NiSaltMe)-PS_low_ (Figure S1b), poly(NiSaltMe)-PS_high_ (Figure S1b′), poly(*meso*-NiSaldMe)-PS_low_ (Figure S2b), and poly(*meso*-NiSaldMe)-PS_high_ (Figure S2b′). The poly(NiSalen)s behaved like a polyphenylene, with the Ni^2+^ ion acting as a bridge between biphenylene moieties.^36^ Importantly, no Ni-centered oxidation, such as Ni^2+^/Ni^3+^, was observed for poly(NiSalen)s at a positive potential range in the moderately electron-donating organic solvent.^33,34,36^ The phenolic salen ligand part belonging to the poly(NiSalen) structure was unequivocally assigned as the primary center of electron release for the poly(NiSalen) film-coated electrodes under such conditions.^33−36^ However, the primary center of electron release can be changed in the presence of electron-donating molecules capable of axial coordination to the Ni^2+^ center of the NiSalen moiety,^50−52^ thus resulting in the electro-oxidation of Ni^2+^ to Ni^3+^.^50,51^ Therefore, the potential-driven transformation of poly(NiSalen)s in 0.2 M aqueous solution of NaOH was recognized as the electrochemically driven Ni(OH)_2_ NP formation based on the change of the primary place of electron release from the phenolic ligand center to the nickel center in a basic electron-donating medium (Scheme 1).

The cleavage of chemical bonds in a molecule by the addition of base with the consequent formation of two bonds with hydroxyl (OH^–^) groups resulting in chemical structure transformation is defined as basic hydrolysis.^53^

During the transformation of polymer films into Ni(OH)_2_-type NPs, the primary target of electron release gradually changed from the phenolic salen center to the Ni center. Consequently, Ni^2+^/Ni^3+^ oxidation peaks were observed at ∼0.5 V, and Ni^3+^ to Ni^2+^ reduction peaks were observed at ∼0.4 V. Thus, the transformation progressed with each potential cycle, and the amount of Ni(OH)_2_-type NPs increased. These ox–red peaks were characteristic of an electrode reaction of poly[SalenNi^2+^(OH)_2_] ⇌ poly(SalenNi^3+^OOH). Notably, the poly(Salen) part was electrochemically inactive when the electron was released from the Ni center. Because of this fact, the above-mentioned reaction is analogical to the well-known reaction of [Ni(OH)_2_ ⇌ NiOOH].^21,22^

The electrochemical transformation of poly(NiSalen) precursors into NPs was manifested by consecutive increases in oxidation and reduction peaks representing Ni^2+^/Ni^3+^ and Ni^3+^/Ni^2+^ processes, indicating the growing number of Ni(OH)_2_-type NPs in the poly(Salen) matrix. The termination of this peak growth was assigned to the complete transformation of the poly(NiSalen) film into [SalenNi^2+^(OH)_2_] NPs. The time needed for the complete transformation of poly(*meso*-NiSaldMe) films into NPs was significantly longer than the time needed for the complete transformation of poly(NiSaltMe) films into NPs (Table 1). This was observed for both types of precursor films, that is, those prepared potentiostatically and potentiodynamically.

The time required to generate NPs from different precursor polymers depends on the polymer film stability (Table 1). The poly(*meso*-NiSaldMe) with very high electrochemical stability needed more time (more CV cycles) for a complete transformation into NPs than poly(NiSaltMe) that featured significantly lower electrochemical stability (Figures 1, 2, S1, and S2).^30^

### Morphological Characterization of Polymer Films and NPs

**Figure 3:**
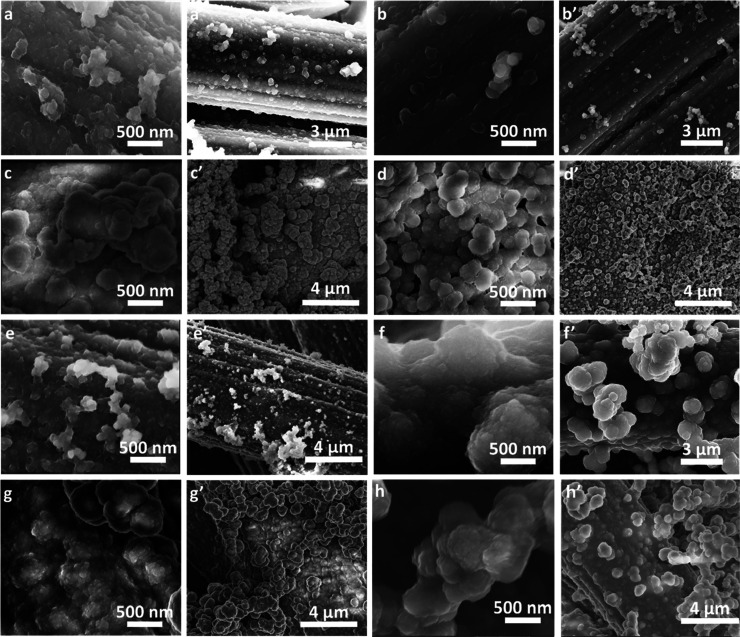
SEM images of precursor films at two magnifications: (a,a′) poly(NiSaltMe)-PD_low′_, (b,b′) poly(NiSaltMe)-PD_high′_, (c,c′) poly(*meso*-NiSaldMe)-PD_low′_, (d,d′) poly(*meso*-NiSaldMe)-PD_high′_, (e,e′) poly(NiSaltMe)-PS_low_, (f,f′) poly(NiSaltMe)-PS_high_, (g,g′) poly(*meso*-NiSaldMe)-PS_low_, and (h,h′) poly(*meso*-NiSaldMe)-PS_high_.

Electropolymerization conditions significantly influenced the morphology of the prepared precursor films (Figure 3). The applied polymerization conditions, that is, PD (Figure 3 a–d′) and PS (Figure 3e–h′) provided distinct morphologies. The CPE fibers were entirely covered with the polymer film (Figure 3) for all samples. This was confirmed by the bare CPE imaging (Figure S3). The poly(NiSaltMe)-PD_low′_ and poly(NiSaltMe)-PD_high′_ films were relatively homogeneous with characteristic rarely scattered bubble-shaped structures (Figure 3a–b′). The poly(*meso*-NiSaldMe)-PD_low′_ film was composed of irregularly stacked globules (Figure 3c,c′), while poly(*meso*-NiSaldMe)-PD_high′_ contained more regular ones (Figure 3d,d′).

Poly(NiSaltMe)-PS_high_ (Figure 3f,f′), poly(*meso*-NiSaldMe)-PS_low_, and poly(*meso*-NiSaldMe)-PS_high_ (Figure 3g–h’) were densely packed films featuring spatially diversified globular structures (cauliflower-like structure). However, poly(NiSaltMe)-PS_low_ (Figure 3e,e′) revealed the smallest size of globules among poly(Salen)s deposited potentiostatically. The characteristic structures of poly(NiSaltMe)-PS_high_ were bigger (Figure 3f) than those of poly(*meso*-NiSaldMe) (Figure 3g,h). The high-magnification images of poly(NiSaltMe)-PS_high_ (Figure 3f′) and poly(*meso*-NiSaldMe)-PS_high_ (Figure 3h′) showed that aggregated globules were layered, that is, big globules were composed of smaller ones.

**Figure 4:**
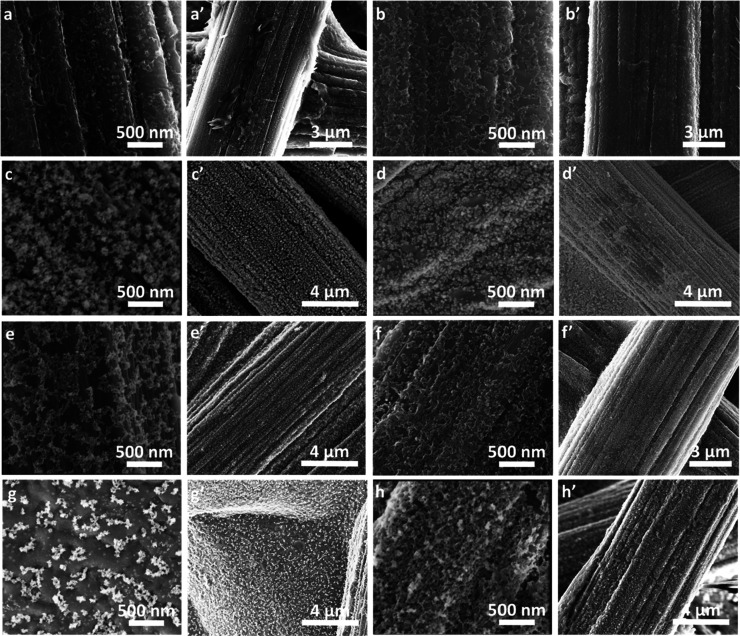
SEM images of Ni(OH)_2_-type NPs derived from (a,a′) poly(NiSaltMe)-PD_low′_, (b,b′) poly(NiSaltMe)-PD_high′_, (c,c′) poly(*meso*-NiSaldMe)-PD_low′_, (d,d′) poly(*meso*-NiSaldMe)-PD_high′_, (e,e′) poly(NiSaltMe)-PS_low_, (f,f′) poly(NiSaltMe)-PS_high_, (g,g′) poly(*meso*-NiSaldMe)-PS_low_, and (h,h′) poly(*meso*-NiSaldMe)-PS_high_.

SEM images of Ni(OH)_2_-type NPs generated from PD- and PS-deposited poly(NiSaltMe) and poly(*meso*-NiSaldMe) films over CPE are shown in Figure 4. As expected, the initial morphologies of precursor films influenced the resulting morphologies of Ni(OH)_2_-type NPs. In contrast to poly(NiSalen) film SEM (Figure 3), the Ni(OH)_2_-type NPs did not show continuous fiber coating but revealed equally distributed NP-coated fibers. NP catalysts derived from poly(NiSaltMe) films (Figure 4a,a′,b,b′,e,e′,f,f′) featured a continuous structure of polymer matrix, arranging NPs in 2D, while those derived from poly(*meso*-NiSaldMe) featured the spatially extended matrix, arranging NPs in 3D (Figure 4c,c′,d,d′g,g′,h,h′). The 3D arrangement of precursor globules (Figure 3c,d,g,h) governed the spatially extended poly(Salen) support structure, arranging NPs in 3D (Figure 4c,d,g,h).

### Electrocatalytic Activity Studies

**Figure 5:**
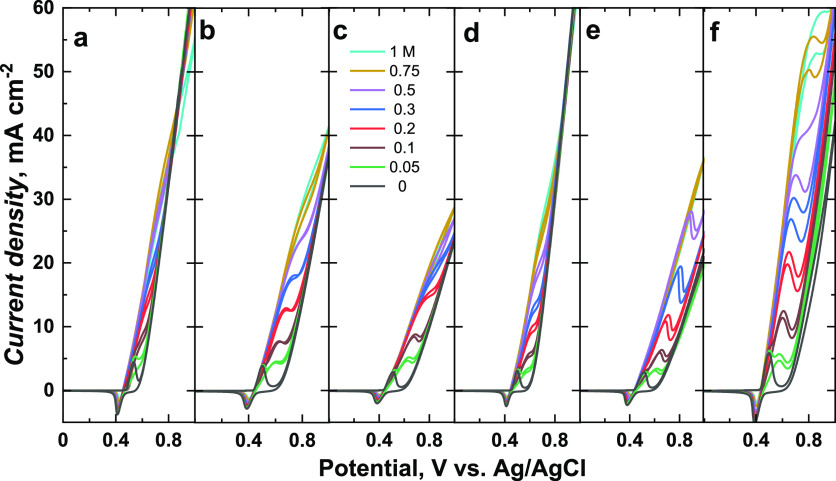
Catalytic CV responses of Ni(OH)_2_-type NPs derived from (a) poly(NiSaltMe)-PD_low_, (b) poly(NiSaltMe)-PD_low′_, (c) poly(NiSaltMe)-PS_low_, (d) poly(NiSaltMe)-PD_high_, (e) poly(NiSaltMe)-PD_high′_, and (f) poly(NiSaltMe)-PS_high_, toward various concentrations of ethanol. CV was performed at 50 mV s^–1^ in 0.2 M NaOH_aq_.

**Figure 6:**
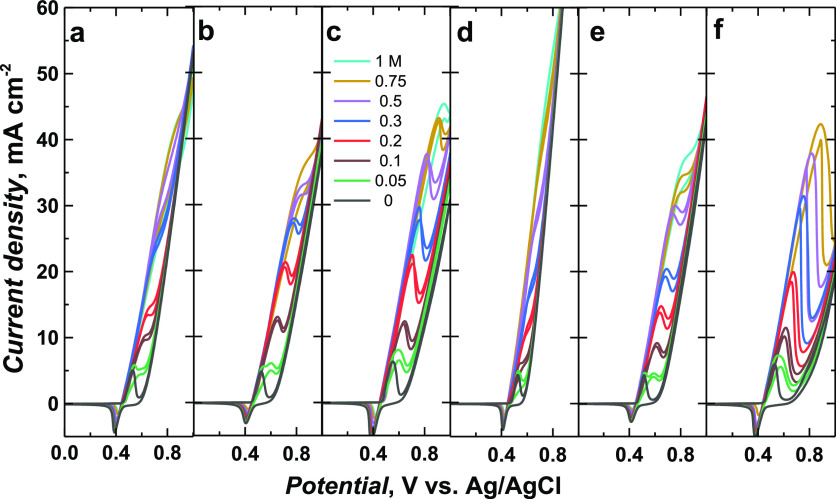
Catalytic CV responses of Ni(OH)_2_-type NPs derived from (a) poly(*meso*-NiSaldMe)-PD_low_, (b) poly(*meso*-NiSaldMe)-PD_low′_, (c) poly(*meso*-NiSaldMe)-PS_low_, (d) poly(*meso*-NiSaldMe)-PD_high_, (e) poly(*meso*-NiSaldMe)-PD_high′_, and (f) poly(*meso*-NiSaldMe)-PS_high_, toward various concentrations of ethanol. The CV curves were recorded at 50 mV s^–1^ in 0.2 M NaOH_aq_.

Figures 5 and 6 showed the catalytic CV responses of Ni(OH)_2_-type NPs derived from poly(NiSaltMe) and poly(*meso*-NiSaldMe), respectively, in the presence of different ethanol concentrations. After the addition of each ethanol concentration, some exciting changes were obtained. The ethanol oxidation during a positive scan is manifested by increasing current density at the potential close to NiOOH formation in a forward scan. This potential was higher than that for NiOOH formation. SEM images helped correlate the catalytic performances of the Ni(OH)_2_-type NPs and their precursor morphologies. The compact films of poly(NiSaltMe) governed the poly(Salen) matrix structure, arranging NPs in 2D, thus resulting in lower catalytic performance (Table 1).

The NPs derived from poly(*meso*-NiSaldMe) were embedded in the poly(*meso*-SaldMe) matrix featuring a spatially extended 3D arrangement, thus resulting in their better catalytic performance. The peak potential associated with the catalytic current shifted to ∼370 mV from its initial value (Figure 6). This shift was minor in comparison to the shift (∼500 mV) observed for NPs derived from the poly(NiSaltMe) (Figure 5).

All Ni(OH)_2_-type NPs demonstrated linear electrocatalytic activity toward ethanol in the concentration range of 0.05–0.5 M. Concentration above this showed catalytic activity saturation. All achievements of ethanol electrocatalysis over different Ni(OH)_2_-type NPs are summarized in Table 1. The main product of electrocatalytic oxidation of ethanol on Ni(OH)_2_-type NPs was recognized as acetic acid (CH_3_COOH).^27^

### Further Characterization of Best-Performing Ni(OH)_2_-Type NPs

Table 1 summarizes the performances of the electrocatalyst for ethanol electro-oxidation. Among all the Ni(OH)_2_-type NPs derived from the poly(*meso*-NiSaldMe) precursors, the NPs derived from poly(*meso*-NiSaldMe)-PS_low_ and poly(*meso*-NiSaldMe)-PS_high_ indicated a minor difference in electrocatalytic activity. However, the polymerization charge used for the deposition of the poly(*meso*-NiSaldMe)-PS_low_ precursor was lower than the polymerization charge used for the deposition of the poly(*meso*-NiSaldMe)-PS_high_ precursor. Because of that, we assumed that Ni(OH)_2_-type NPs derived from poly(*meso*-NiSaldMe)-PS_low_ were better than those of NPs derived from poly(*meso*-NiSaldMe)-PS_high._ Ultimately, by analyzing the polymerization charges of precursors and the catalytic performance of Ni(OH)_2_-type NPs derived from them, we selected NP catalysts derived from poly(NiSaltMe)-PS_high_ and poly(*meso*-NiSaldMe)-PS_low_ for further studies (Table 1).

### STEM Images of Ni(OH)_2_-Type NPs Derived from Poly(Salen) Films

**Figure 7:**
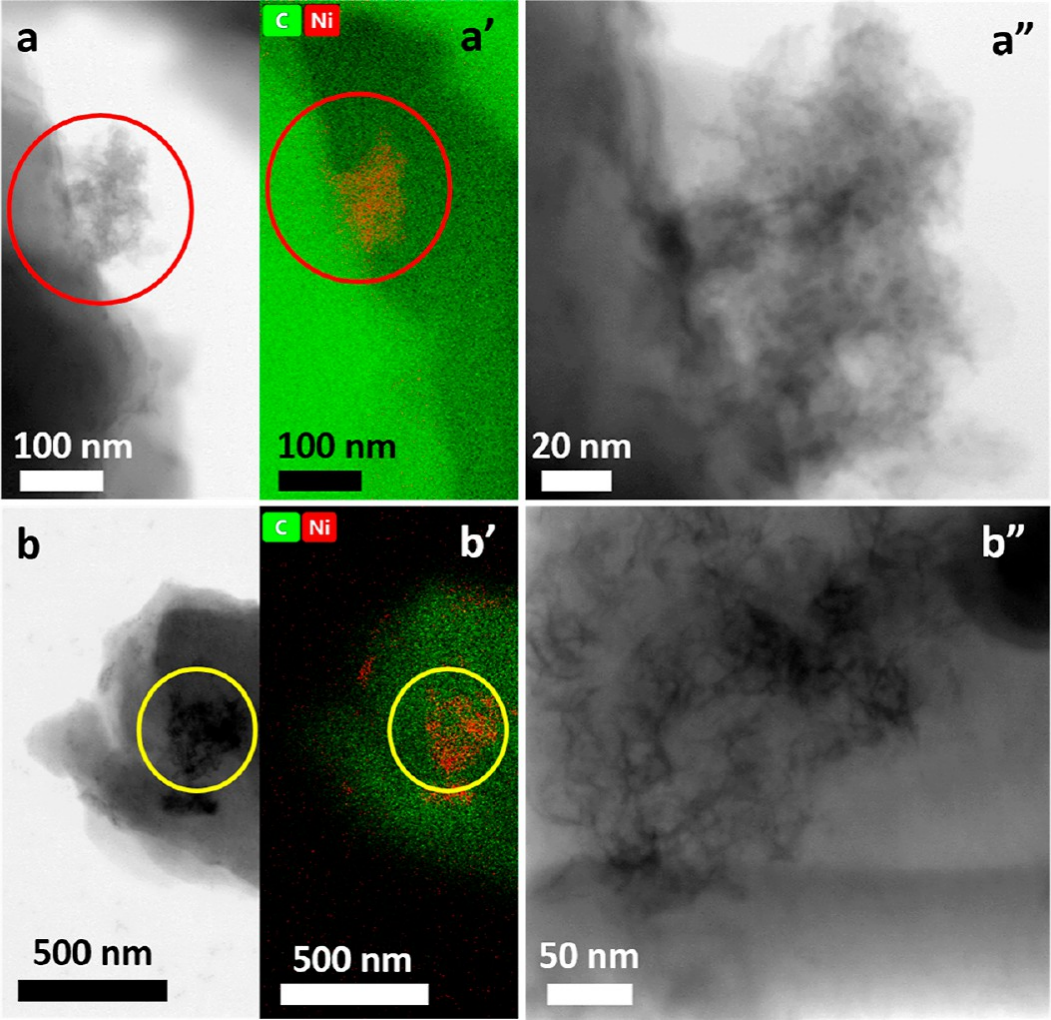
STEM images (a,a″,b,b″) and EDX mapping (a′,b′) of Ni(OH)_2_-type NPs derived from (a,a′,a″) poly(NiSaltMe)-PS_high_ and (b,b′,b″) poly(*meso*-NiSaldMe)-PS_low_.

STEM images of Ni(OH)_2_-type NPs derived from poly(NiSaltMe)-PS_high_ (Figure 7a) and poly(*meso*-NiSaldMe)-PS_low_ (Figure 7b) with a fragment of the CPE substrate removed during scratching are shown in Figure 7. EDS elemental profiling (Figure 7a′,b′, circled area) confirmed the coexistence of main elements Ni and C, thus proving the presence of Ni(OH)_2_-type NPs over the carbon substrate. Moreover, it revealed that the Ni(OH)_2_-type NPs are surrounded by poly(Salen) matrixes. The observed distances between NPs were bigger for NPs derived from poly(*meso*-NiSaldMe)-PS_low_ (Figure 7b′) than for NPs derived from poly(NiSaltMe)-PS_high_ (Figure 7a′). Figure 7a″,b″ showed a magnified view of Figure 7a,b focused on NPs and confirmed different spatial distributions of polymer matrixes determining the arrangement of NPs. The poly(*meso*-SaldMe)-PS_low_ matrix indicated a higher spatial distribution than that of the poly(SaltMe)-PS_high_ matrix. The Ni(OH)_2_-type NPs in spatially extended non-conducting poly(Salen) matrixes (in NaOH_aq_) prevented the permanent adsorption of counterions during doping and dedoping as well as the substrate and product of the catalytic reaction.

### XPS Characterization of Ni(OH)_2_-Type NPs Derived from Poly(NiSalen) Films

**Figure 8:**
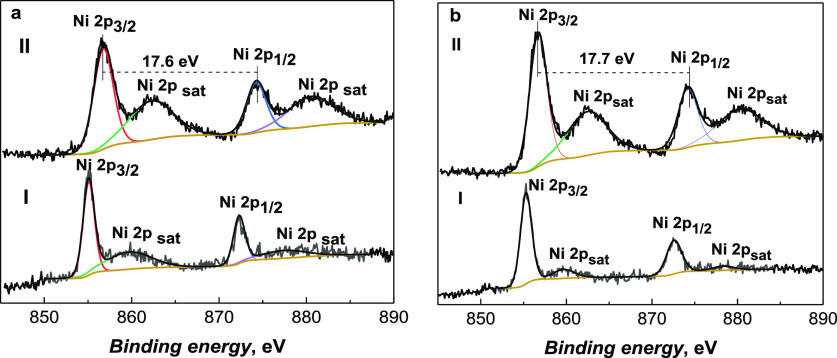
Ni 2p XPS spectra of (a I) poly(*meso*-NiSaldMe)-PS_low_ and (a II) NPs derived from poly(*meso*-NiSaldMe); (b I) poly(NiSaltMe)-PS_high_ and (b II) NPs generated from poly(NiSaltMe).

XPS was used to characterize the differences in the chemical nature of Ni^2+^ species present in both the precursor polymer films and NP samples. Figures 8 and S4 summarize the Ni^2+^ (Ni 2p) HR spectra of poly(Salen)s and NPs derived from them. The Ni 2p spectra of as-prepared Ni(OH)_2_ NPs derived from both poly(Salen)s exhibit the binding energies of Ni 2p_3/2_ and Ni 2p_1/2_ at ∼856 and ∼874 eV, respectively (Figures 8 and S4). The binding energy difference between Ni 2p_3/2_ and Ni 2p_1/2_ peaks varied from ∼17.5 to 17.6 eV, thus confirming the presence of Ni(OH)_2_-type NPs.^12,54^ Moreover, the satellite peak appearance implicated the presence of a high-spin divalent state of Ni^2+^ in the Ni(OH)_2_ NP samples and confirmed the loss of square-planar geometry of Ni^2+^.^55^ The XPS signal detected binding energies of Ni 2p_3/2_ and Ni 2p_1/2_ in polymer spectra, which were slightly different from those of NPs. For instance, in poly(*meso*-NiSaldMe)-PS_low_, the binding energies of Ni 2p_3/2_ and Ni 2p_1/2_ were 855.3 and 872.5 eV, respectively, while for the corresponding NP sample, they were found to be shifted to 856.6 and 874.2 eV, respectively (Figures 8, and S4). The results of O 1s (Figure S5) and C 1s (Figure S6) XPS spectra for poly(NiSaltMe) and poly(*meso*-NiSaldMe) and NPs generated from those poly(Salen) films are discussed in the Supporting Information.

### Retention of Catalytic Properties and Regeneration of Nanoparticles

In order to investigate the retention of catalytic activity of Ni(OH)_2_-type NPs, the voltammetric multi-cyclic curves were recorded in the presence of 0.3 M ethanol (Figure S7). Additionally, SEM images (Figure S8) and XPS spectra (Figure S9) were registered after these multi-cyclic CV responses of Ni(OH)_2_-type NPs derived from poly(NiSaltMe)-PS_high_ and poly(*meso*-NiSaldMe)-PS_low_.

**Figure 9:**
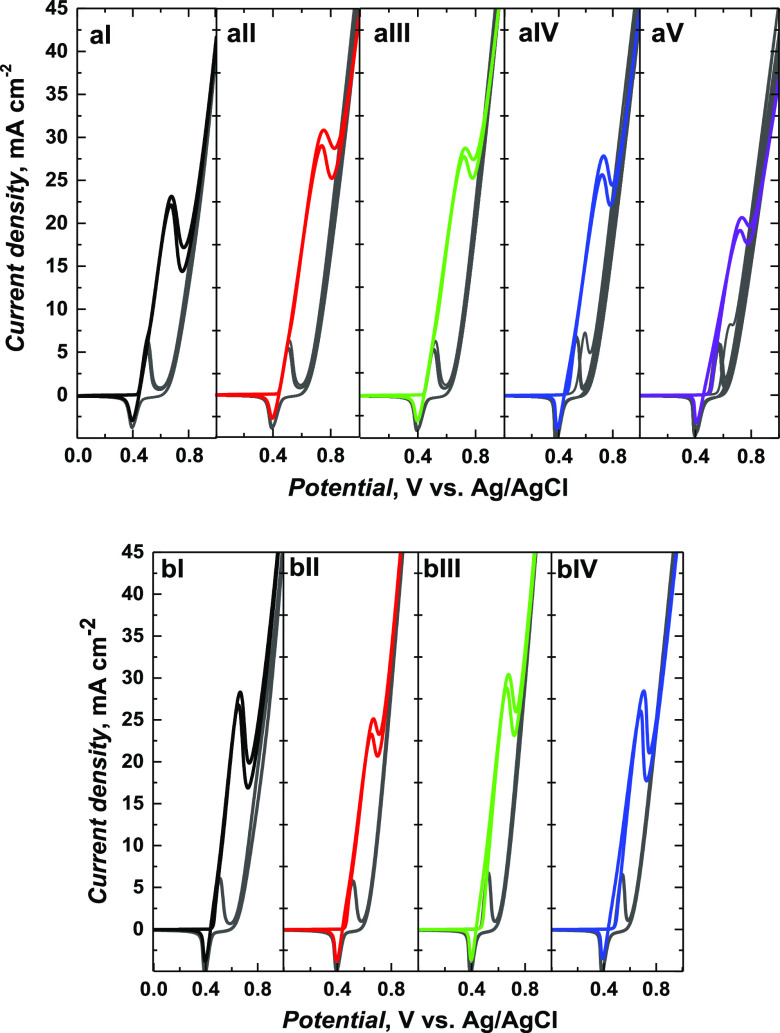
Electrocatalytic currents obtained over (I) freshly prepared as well as (II–IV) regenerated NPs with a scan rate of 50 mV s^–1^ in 0.2 M NaOH_aq_ in the presence of 0.3 M ethanol for NPs derived from (a) poly(NiSaltMe)-PS_high_ and (b) poly(*meso*-NiSaldMe)-PS_low_. (a V) Electrocatalytic current measured after 1 month of storage.

Repeated CV measurements did not alter the electro-oxidation current (Figure S7). Furthermore, multiple regenerations of Ni(OH)_2_-type NPs were possible (Figure 9a,b). The catalytic currents observed after each regeneration were of the same order of magnitude (Figure 9a,b), and no catalytic poisoning effect was observed, as reported by others. The amount of Ni^2+/^Ni^3+^ active centers remaining on the electrode surface was slightly decreased (Figure 9) after its reaction with ethanol, thus indicating the auto-regeneration of Ni^3+^/Ni^2+^ species in the reverse cycle. Because of that, retention of electrocatalytic currents was observed during multiple CV cycles (Figure S7). Moreover, the catalytic current observed after 1 month of storage (Figure 9a V) indicated minor changes in comparison to freshly prepared Ni(OH)_2_-type NPs (Figure 9a I).

SEM images registered after electro-oxidation of ethanol on Ni(OH)_2_-type NPs derived from poly(NiSaltMe)-PS_high_ (Figure S8a) and poly(*meso*-NiSaldMe)-PS_low_ (Figure S8b) are shown in Figure S8. Poly(*meso*-NiSaldMe)-PS_low_ (Figure S8b) did not show any significant changes in its morphology. NPs derived from poly(NiSaltMe)-PS_high_ (Figure S8a) display a slight change in morphology, presumably caused by adsorption of products on the catalyst. However, active centers were easily regenerated in 0.2 M NaOH_aq_. Furthermore, no differences in the chemical nature of Ni^2+^ species were observed in XPS spectra registered after ethanol electro-oxidation on both NP catalysts (Figure S9). The Ni 2p spectra after ethanol electro-oxidation exhibit the binding energies of Ni 2p_3/2_ and Ni 2p_1/2_ at ∼857.0 and ∼874.6 eV (Figure S9a) for the poly(NiSaltMe)-PS_high_-derived catalyst and those of ∼856.3 and ∼874.0 eV (Figure S9b) for the poly(*meso*-NiSaldMe)-PS_low_-derived catalyst, respectively. All of these observations support the fact that Ni(OH)_2_ derived from poly(*meso*-NiSaldMe) and poly(NiSaltMe) are resistant to poisoning effects and because of that can be used multiple times.

### Electrochemical Characterization of NPs Derived from Poly(NiSaltMe)-PS_high_ and Poly(*meso*-NiSaldMe)-PS_low_

Two samples of Ni(OH)_2_-type NPs derived from poly(NiSaltMe)-PS_high_ and poly(*meso*-NiSaldMe)-PS_low_ indicating the best electrocatalytic performance (Table 1) were selected for further electrochemical studies of poly[SalenNi^2+^ (OH)_2_] ⇌ poly(SalenNi^3+^OOH) charge transfer across CPE and NP catalyst materials and associated mass transport processes in 0.2 M NaOH_aq_ by involving multiple scan rate CV and constant potential EIS experiments.

**Figure 10:**
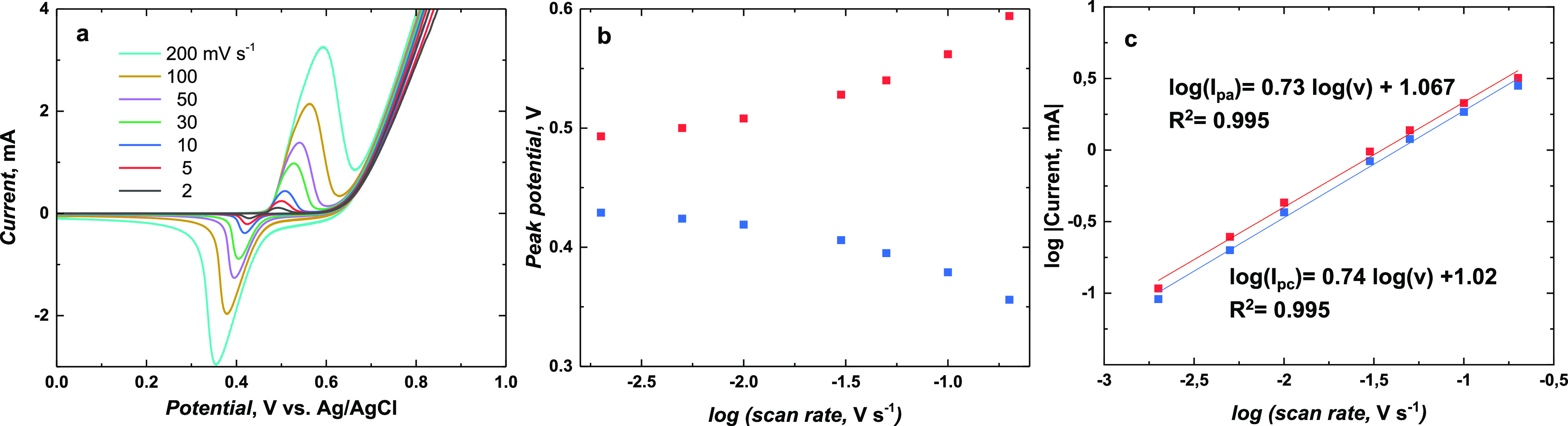
(a) CV curves of Ni(OH)_2_-type NPs derived from poly(NiSaltMe)-PS_high_ performed at 2, 5, 10, 30, 50, 100, and 200 mV s^–1^ scan rates in 0.2 M NaOH_aq_. (b) Anodic (red curve) and cathodic (blue curve) peak potentials vs logarithm of the scan rate. (c) Logarithm of the anodic (red curve) and cathodic (blue curve) peak current dependence on the logarithm of the scan rate.

**Figure 11:**
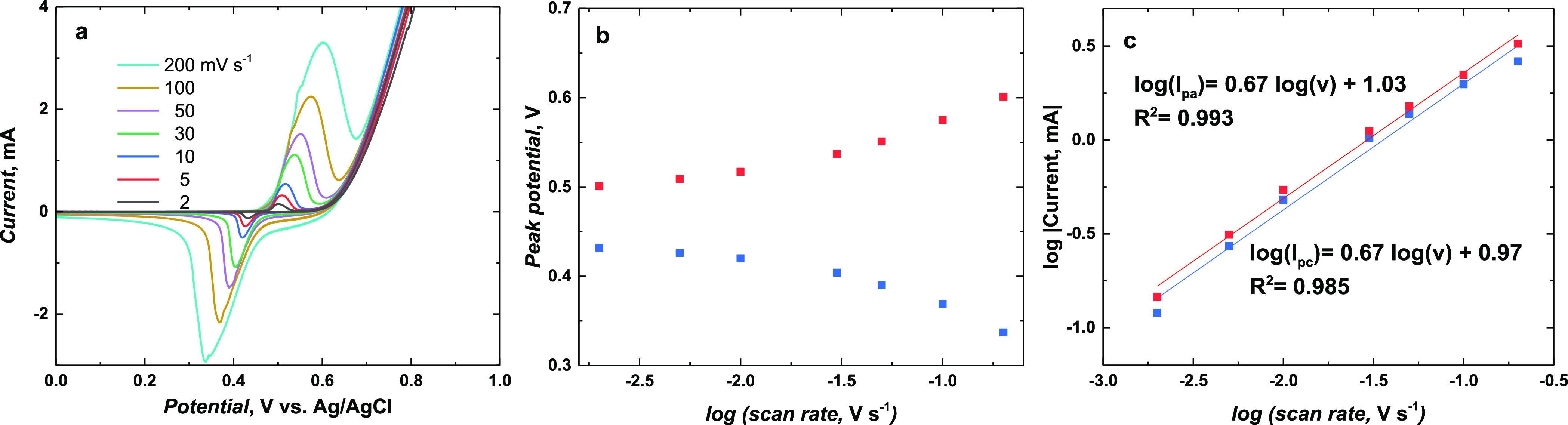
(a) CV curves of Ni(OH)_2_-type NPs derived from poly(*meso*-NiSaldMe)-PS_low_ registered at 2, 5, 10, 30, 50, 100, and 200 mV s^–1^ in 0.2 M NaOH_aq_. (b) Anodic (red curve) and cathodic (blue curve) peak potentials vs logarithm of the scan rate. (c) Logarithm of the anodic (red curve) and cathodic (blue curve) peak current dependence on the logarithm of the scan rate.

The influence of the scan rate change on the electrochemical behavior of Ni(OH)_2_-type NPs derived from poly(NiSaltMe)-PS_high_ (Figure 10a) and poly(*meso*-NiSaldMe)-PS_low_ (Figure 11a) was investigated in 0.2 M NaOH_aq_ at 2, 5, 10. 30, 50, 100, and 200 mV s^–1^. Multiple scan rate CV responses exhibited one anodic and one cathodic peak, indicating one-electron oxidation and one-electron reduction occurrences, respectively.

Charge transfer is the controlling step when the rate of charge transport within the electroactive species confined to the electrode is the slowest process and the redox equilibrium does not prevail. Thus, in such a case, for the multiple scan rate experiment, the separation between the anodic and cathodic CV peaks increases proportionally together with a decrease in peak currents because the rate of charge transfer becomes slower. None of the redox-active materials can serve as a potential electrocatalyst if their electrochemical behavior in the supporting electrolyte medium is controlled by slow charge transfer. This behavior was not observed for Ni(OH)_2_-type NPs distributed in the poly(SaltMe) or poly(*meso*-SaldMe) matrix deposited over the CPE (Tables S1 and S2) (Figures 10 and 11). The charge-transfer processes within both poly(NiSaltMe)-PS_high_- and poly(*meso*-NiSaldMe)-PS_low_-derived NPs were fast.

The peak potentials of NPs derived from poly(NiSaltMe)-PS_high_ did not change significantly with the change in the scan rate up to 10 mV s^–1^ (Figure 10a). However, an increase in the peak potential separation was observed from 30 to 200 mV s^–1^ (Figure 10b). The linear dependences of the logarithm of the anodic (red curve) and cathodic (blue curve) peak currents on the logarithm of the scan rate, with the slopes ∼0.73 and 0.74, respectively, suggest that the oxidation and reduction of poly(NiSaltMe)-PS_high_-derived NPs are under finite diffusion control,^56,57^ indicating that the thickness of the poly(NiSaltMe)-PS_high_-derived NPs was lower than or equal to the thickness of the diffusion layer at studied scan rates. However, the onset of changes in the peak potential observed for scan rates higher than 50 mV suggests the beginning of a change from a thin layer regime to a semi-infinite diffusion rate control.^56,57^

The linear dependences of the logarithm of the anodic (red curve) and cathodic (blue curve) peak currents on the logarithm of the scan rate obtained for poly(*meso*-NiSaldMe)-PS_low_ derived NPs with a slope of ∼0.67 suggested that oxidation and reduction were close to entering the semi-infinite diffusion rate control. The relatively significant peak potential separations observed for Ni(OH)_2_-type NPs derived from poly(*meso*-NiSaldMe)-PS_low_ (Table S2 and Figure 11a) were most likely induced by the Ohmic drop caused by the spatially extended 3D construction of non-conducting in aqueous condition matrix of poly(*meso*-SaldMe). Moreover, the observed peaks were broader (Figure 11a) than those observed for Ni(OH)_2_-type NPs derived from poly(NiSaltMe)-PS_high_ (Figure 10a). This indicated the occurrence of the repulsive interaction between components^58^ of the NP material derived from poly(*meso*-NiSaldMe)-PS_low_. Furthermore, the redox equilibrium of poly[SalenNi^2+^ (OH)_2_] ⇌ poly(SalenNi^3+^OOH) has prevailed for both poly(NiSaltMe)-PS_high_- and poly(*meso*-NiSaldMe)-PS_low_-derived NP electrodes, that is, their behavior was Nernstian.^59^ The analysis described above for NPs derived from poly(NiSaltMe)-PS_high_ and poly(*meso*-NiSaldMe)-PS_low_ proved that one-electron exchange reversible electrochemical processes of poly[SalenNi^2+^ (OH)_2_] ⇌ poly(SalenNi^3+^OOH) in 0.2 M NaOH_aq_ were fast and diffusion-controlled.

To get closer insights into the differences observed in the electrochemical behavior of Ni(OH)_2_-type NPs derived from poly(NiSaltMe)-PS_high_ (Table 2 and Figure 10a) and poly(*meso*-NiSaldMe)-PS_low_ (Table 3 and Figure 11a) and to study the influence of differently structured non-conducting poly(Salen) matrixes in aqueous conditions, the counterion accessible area for both NPs was calculated, *A*_CI_. The physical meaning of this value reflects the material surface development in relation to the geometric surface.
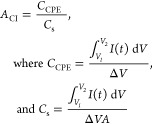
1

**Table 2:** *A*_CI_ Values Calculated from the Multi-scan Rate Experiment Performed for Ni(OH)_2_-Type NPs Derived from Poly(NiSalen) Films in 0.2 M NaOH_aq_

	*A*_CI_ (cm^2^)	
*v* (mV s^–1^)	NPs derived from poly(NiSaltMe)-PS_high_	NPs derived from poly(*meso*-NiSaldMe)-PS_low_
2	0.180	0.197
5	0.150	0.164
10	0.128	0.146
30	0.057	0.064
50	0.036	0.041
100	0.019	0.021
200	0.010	0.011

**Table 3:** Surface Concentrations of Ni^3+^ Oxy-Hydroxide Centers Determined from the Multi-scan Rate CV Experiments within Three Consecutive Cycles Performed for Ni(OH)_2_-Type NPs Derived from Poly(NiSalen) Films in 0.2 M NaOH_aq_ in the Absence and Presence of 0.3 M Ethanol

	Γ of NPs derived from poly(NiSaltMe)-PS_high_ (μmol cm^–2^)			Γ of NPs derived from poly(NiSaltMe)-PS_high_ with ethanol (μmol cm^–2^)			Γ of NPs derived from poly(*meso*-NiSaldMe)-PS_low_ (μmol cm^–2^)			Γ of NPs derived from poly(*meso*-NiSaldMe)-PS_low_ with ethanol (μmol cm^–2^)		
*v* (mV s^–1^)	cycle 1	cycle 2	cycle 3	cycle 1	cycle 2	cycle 3	cycle 1	cycle 2	cycle 3	cycle 1	cycle 2	cycle 3
2	0.093	0.086	0.087	0.012	0.013	0.012	0.094	0.090	0.093	0.019	0.018	0.019
5	0.082	0.083	0.083	0.024	0.024	0.021	0.093	0.088	0.090	0.026	0.024	0.025
10	0.082	0.080	0.082	0.027	0.027	0.028	0.089	0.088	0.088	0.031	0.033	0.032
30	0.081	0.078	0.077	0.037	0.035	0.036	0.081	0.080	0.082	0.041	0.040	0.040
50	0.074	0.075	0.074	0.040	0.039	0.038	0.077	0.077	0.075	0.043	0.043	0.043
100	0.071	0.071	0.073	0.044	0.043	0.043	0.074	0.075	0.076	0.045	0.046	0.045
200	0.066	0.066	0.065	0.045	0.045	0.045	0.069	0.069	0.070	0.048	0.048	0.048

*A*_CI_ was calculated according to eq 1, whereas *C*_CPE_ and *C*_s_ stand for the capacitance of the CPE and specific capacitance (capacitance divided by the geometric electrode area, *A*) of Ni(OH)_2_-type NPs derived from both polymers, respectively, and determined from the discharging part of CV curves registered in 0.2 M NaOH_aq_ at different scan rates. The obtained *A*_CI_ values are summarized in Table 2.

Diffusion control of Ni^2+^ ⇌ Ni^3+^ processes observed for both Ni(OH)_2_-type NPs derived from poly(NiSaltMe)-PS_high_ and poly(*meso*-NiSaldMe)-PS_low_ in 0.2 M NaOH_aq_ is reflected in the *A*_CI_ values (Table 2). These values are the highest for the lowest scan rate and the lowest for the highest scan rate. It is because OH^–^ counterions cannot keep up with the fast charge transfer of Ni^2+^ ⇌ Ni^3+^ at higher scan rates. The *A*_CI_ values determined for NPs derived from poly(*meso*-NiSaldMe)-PS_low_ from the multi-scan rate experiment registered in 0.2 M NaOH_aq_ were higher than those determined for NPs prepared from poly(NiSaltMe)-PS_high_ (Table 2). This explains why NPs derived from poly(*meso*-NiSaldMe)-PS_low_ are better for electrocatalytic purposes than NPs derived from poly(NiSaltMe)-PS_high_. Diffusion of OH^–^ counterions maintaining the charge generated during fast Ni^2+^ ⇌ Ni^3+^ processes is less hindered for NPs derived from poly(*meso*-NiSaldMe)-PS_low_ than NPs derived from poly(NiSaltMe)-PS_high._ We anticipate that the spatially extended 3D construction of the poly(*meso*-SaldMe)-PS_low_ matrix surrounding Ni(OH)_2_ NPs favors such electrochemical behavior.

**Figure 12:**
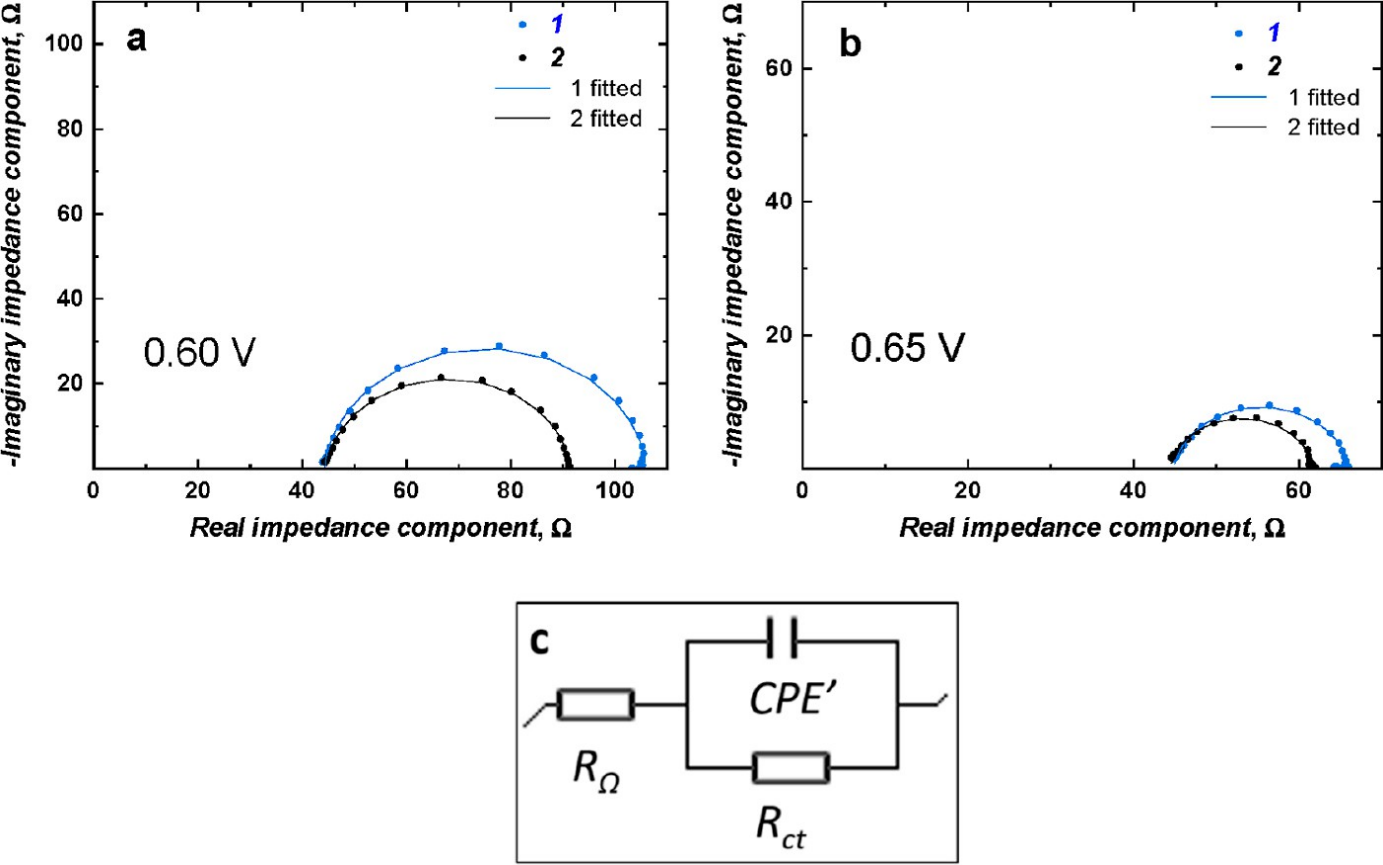
Comparative Nyquist plots for (1) NPs derived from poly(NiSaltMe)-PS_high_ and (2) NPs derived from poly(*meso*-NiSaldMe)-PS_low_. EIS measurements were registered in 0.2 M NaOH_aq_ for constant potentials of (a) 0.60 and (b) 0.65 V. Before the EIS measurements, each electrode was equilibrated by applying the selected potential for 2 min (Figure S10). After that time, the current reached equilibrium and EIS measurements were then performed with the voltage amplitude of 10 mV in the frequency range of 10 kHz to 10 mHz. (c) Modified Randles–Ershler equivalent electric circuit composed of *R*_Ω_ (Ohmic resistance), CPE′ (constant phase element), and *R*_ct_ (charge-transfer resistance) electrical elements was used for fitting the individual electrical circuit elements to the experimental data.

EIS experiments were employed to compare the rate of charge transfer occurring in NPs derived from poly(*meso*-NiSaldMe)-PS_low_ and NPs derived from poly(NiSaltMe)-PS_high_ at 0.6 and 0.65 V vs Ag/AgCl—potentials favoring electro-oxidation of poly[SalenNi^2+^ (OH)_2_] to poly(SalenNi^3+^OOH) in 0.2 M NaOH_aq_ (Figure 12).

The characteristic values of *R*_Ω_, *C*_dl_, and *R*_ct_ (Table S4) obtained by fitting experimental data (Figure 12a,b) to the equivalent circuit (Figure 12c) describe characteristic electrochemical processes occurring for both types of NPs derived from poly(*meso*-NiSaldMe)-PS_low_ and NPs derived from poly(NiSaltMe)-PS_high_ at 0.6 and 0.65 V vs Ag/AgCl. The *R*_Ω_, in the Nyquist plots (Figure 12a,b), manifests itself at high frequencies as a first point on the real impedance component axis. It is equivalent to the sum of resistances belonging to the electrolyte solution, NP materials, and electrical connections. The subsequent semicircles in the Nyquist plots (Figure 12a,b) are characteristic of the charge-transfer processes, that is, heterogeneous redox Ni^2+^/Ni^3+^reactions occurring at the interface of CPE|NPs surrounded by the poly(SaldMe) matrix simultaneously with the double-layer charging. Therefore, *R*_ct_ and *C*_dl_ are connected in parallel. The *C*_dl_ of both NP-based materials was replaced by the CPE′ because those materials were not behaving like an ideal capacitor. The values of *R*_ct_ are characteristic of probing the rate of charge transfer.^60,61^ The *R*_ct_ values are higher for NPs derived from poly(NiSaltMe)-PS_high_ than those observed for NPs derived from poly(*meso*-NiSaldMe)-PS_low_ (Table S4). Furthermore, *C*_dl_ values obtained for NPs derived from poly(*meso*-NiSaldMe)-PS_low_ are higher than those for NPs derived from poly(NiSaltMe)-PS_high_ (Table S4). It means that the rates of charge transfer in 0.2 M NaOH_aq_ for NPs derived from poly(*meso*-NiSaldMe)-PS_low_ at 0.6 and 0.65 V versus Ag/AgCl are higher than those observed for NPs derived from poly(NiSaltMe)-PS_high_ under similar experimental conditions. Moreover, higher values of *C*_dl_ and lower values of *R*_ct_ obtained for NPs derived from poly(*meso*-NiSaldMe)-PS_low_ confirmed the effective distribution of Ni(OH)_2_-type NPs in the spatially extended 3D matrix of poly(*meso*-SaldMe)-PS_low_, facilitating faster and more effective charge transfer.

### Electrocatalytic Oxidation Reaction of Ethanol on Ni(OH)_2_-Type NPs Derived from Poly(NiSaltMe)-PS_high_ and Poly(*meso*-NiSaldMe)-PS_low_

**Figure 13:**
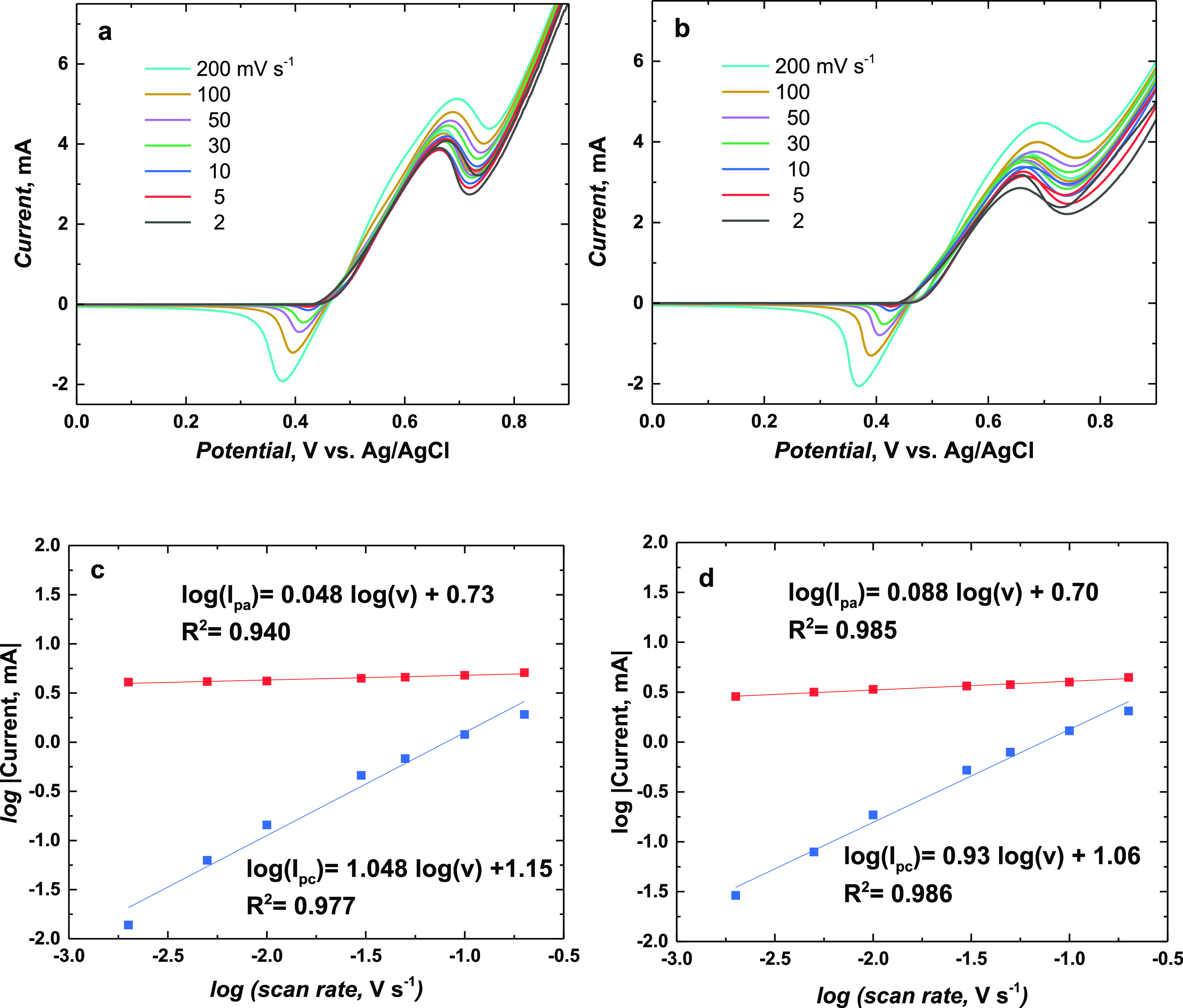
CV curves of Ni(OH)_2_-type NPs derived from (a) poly(NiSaltMe)-PS_high_ and (b) poly(*meso*-NiSaldMe)-PS_low_ at 2, 5, 10, 30, 50, 100, and 200 mV s^–1^ registered in a solution containing 0.2 M NaOH_aq_ and 0.3 M ethanol.

In order to analyze the electrocatalytic oxidation reaction (EOR) of ethanol on Ni(OH)_2_-type NPs derived from poly(NiSaltMe)-PS_high_ and poly(*meso*-NiSaldMe)-PS_low_, the surface concentrations (Γ)^62^ of Ni^2+^ produced in the reduction of oxy-hydroxide centers (Ni^3+^OOH) were determined from the cathodic parts of the multi-scan rate CV experiments within three consecutive cycles performed in 0.2 M NaOH_aq_ in the presence and absence of 0.3 M ethanol (Figure 13). The Γ values were determined according to eq 2.
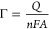
2where *Q* is the cathodic charge, *n* is the number of exchanged electrons, *F* is the Faraday constant, and *A* is the geometric area of the electrode. The obtained Γ values are summarized in Table 3.

Herein, we studied that ethanol electro-oxidation catalyzed by Ni(OH)_2_-type NPs derived from poly(NiSalen)s undergoes an EC anodic-oxidative mechanism.^63^ As a consequence of the catalytic reaction, the number of oxidized Ni^3+^ species is reduced. Therefore, the whole process results in a decrease in the cathodic peak current *I*_pc_ when compared to the fast diffusion-controlled electrochemical process observed for NPs derived from poly(NiSalen)s in NaOH_aq_. The analyzed process can be described as an EC mechanism featuring two parts (electrochemical and chemical) occurring at overlapping potentials, that is, (i) material electrochemical reaction: poly[SalenNi^2+^ (OH)_2_] ⇌ poly(SalenNi^3+^OOH) (electrochemical part of the process) and (ii) ethanol oxidation to acetic acid on poly(SalenNi^3+^OOH) centers (chemical part of the process). The current increase originating from the poly(SalenNi^3+^OOH) + CH_3_CH_2_OH → CH_3_COOH + poly[SalenNi^2+^ (OH)_2_] manifests catalytic occurrence.

The easily accessible Ni^3+^ oxy-hydroxide centers (Ni^3+^OOH) are necessary for a catalytic process to proceed, and for this reason it is essential to fabricate the catalyst with the highest catalytic activity. Moreover, they should be able to regenerate in NaOH_aq_. Changing the scan rate of the CV alters the time taken between the peak cathodic and peak anodic potentials: decreasing the scan rate allows more time for the chemical part of the process, resulting in a decreased concentration of Ni^2+^ arising from the reduction of accessible Ni^3+^ centers to Ni^2+^ in a cathodic CV scan (Figure 13). This results in greater irreversibility in the CV response and a decrease in the *I*_pa_/*I*_pc_ ratio (Table S3).

Because of that, Γ values determined for NP catalysts in the presence of 0.3 M ethanol are lower than those determined for NP catalysts in 0.2 M NaOH_aq_ without ethanol (Table 3). More importantly, these centers are autocatalytically re-constructed in the subsequent cycles (Table 3). This was proven by Γ values obtained in the presence of ethanol in three consecutive catalytic cycles. Even in the presence of ethanol, a minor change in Γ values was observed. The amount of catalytically active Ni^3+^OOH centers was slightly higher for Ni(OH)_2_-type NPs derived from poly(*meso*-NiSaldMe)-PS_low_ than for those derived from poly(NiSaltMe)-PS_high_ (Table 3). This indicated why the catalytic performance of Ni(OH)_2_-type NPs derived from poly(*meso*-NiSaldMe)-PS_low_ was better (Table 1, Figure 9).

The electrochemically active surface area (*A*_ECSA_) of NPs derived from poly(NiSaltMe)-PS_high_ and poly(*meso*-NiSaldMe)-PS_low_ was estimated with the oxalate method (Figure S11, Supporting Information). These values supported surface concentrations of Ni^3+^ oxy-hydroxide where Ni(OH)_2_-type NPs derived from poly(*meso*-NiSaldMe)-PS_low_ indicate a higher amount of active redox species.

**Figure 14:**
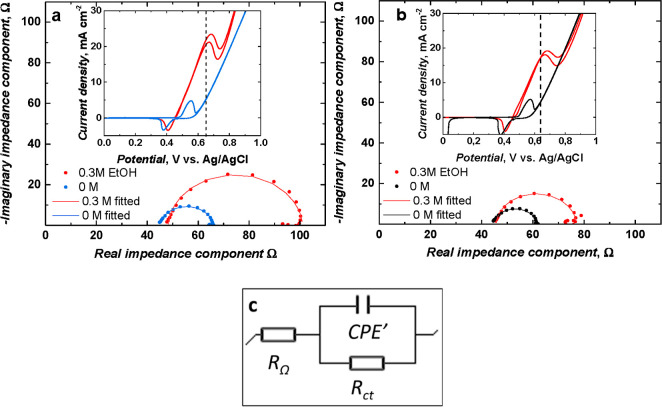
Nyquist plots for Ni(OH)_2_-type NPs derived from (a) poly(NiSaltMe)-PS_high_ and (b) poly(*meso-*NiSaldMe)-PS_low_ prepared based on EIS measurements conducted in 0.2 M NaOH_aq_ in the absence and presence of 0.3 M ethanol at a potential of 0.65 V. Before the EIS measurements, each electrode was equilibrated by applying the selected potential for 2 min. After that time, the current reached equilibrium, and EIS measurements were performed with the voltage amplitude of 10 mV in the frequency range of 10 kHz to 10 mHz. Insets display the CV curve of Ni(OH)_2_-type NPs derived from (a) poly(NiSaltMe)-PS_high_ and (b) poly(*meso-*NiSaldMe)-PS_low_ at 50 mV s^–1^ in 0.2 M NaOH_aq_ in the absence and presence (red) of 0.3 M ethanol. (c) Modified Randles–Ershler equivalent electric circuit composed of *R*_Ω_, CPE′, and *R*_ct_ electrical elements used for fitting of the individual electrical circuit element values to the experimental data.

The anodic current for ethanol oxidation increased slowly with an increase in scan rates. The total process associated with electrocatalysis, that is, poly(SalenNi^3+^OOH) + CH_3_CH_2_OH → CH_3_COOH + poly[SalenNi^2+^ (OH)_2_] involving the chemical reaction of ethanol oxidation at catalytic centers of both Ni(OH)_2_-type NPs derived from poly(NiSaltMe)-PS_high_ and poly(*meso*-NiSaldMe)-PS_low_ (Figure 13), is slower than the electrochemical process of poly[SalenNi^2+^ (OH)_2_] ⇌ poly(SalenNi^3+^OOH) occurring for these NPs in pure 0.2 M NaOH_aq_. Moreover, EIS experiments conducted for Ni(OH)_2_-type NPs derived from poly(NiSaltMe)-PS_high_ and poly(*meso*-NiSaldMe)-PS_low_ in 0.2 M NaOH_aq_ in the absence and presence of 0.3 M ethanol at a constant potential of 0.65 V vs Ag/AgCl confirmed the above observation. The *R*_ct_ values fitted to experimental data obtained at 0.65 V in the presence of 0.3 M ethanol (Figure 14) for both types of NPs were higher than those observed in pure 0.2 M NaOH_aq_ (Table S4), thus confirming that the charge-transfer processes were slower during the electrocatalytic oxidation of ethanol.

Moreover, the catalytic charge-transfer process was faster for the NP catalyst derived from poly(*meso*-NiSaldMe)-PS_low_ (Figure 14b) than for the NP catalyst derived from poly(NiSaltMe)-PS_high_ (see *R*_ct_ values in Table S4). The increase in the *R*_Ω_ value observed for Ni(OH)_2_-type NPs derived from poly(NiSaltMe)-PS_high_ in 0.3 M ethanol (Figure 14a, red curve) suggests that the substrate (ethanol) adsorbs strongly on this catalyst during electrocatalysis. The presented EIS results obtained for both types of NPs at 0.65 V in the presence of 0.3 M ethanol confirmed that Ni(OH)_2_-type NPs derived from poly(*meso*-NiSaldMe)-PS_low_ possess better electrocatalytic performance.

Furthermore, the multi-scan rate CV experiment indicated that the time window for the ethanol oxidation process at higher scan rates becomes narrow; in this situation, facile electron transfer between ethanol and Ni(OH)_2_-type NPs (that control the whole electrocatalytic process) becomes less probable. This charge transfer is the controlling step in this case, and the redox equilibrium does not prevail. In the multiple scan rate experiment, the separation between the cathodic and anodic CV peaks increases for the lower values of peak currents; because of that, the rate of charge transfer between ethanol and Ni(OH)_2_-type NPs becomes slower at higher scan rates (Figure 13a,b). Concluding, the slowest process of electron transfer (between ethanol and Ni(OH)_2_-type NPs) is the controlling step of the described electrocatalysis on Ni(OH)_2_-type NPs derived from poly(NiSalen)s.

In order to understand the better catalytic performance of the Ni(OH)_2_-type NP catalyst derived from poly(*meso*-NiSaldMe)-PS_low_ in comparison to NPs derived from poly(NiSaltMe)-PS_high_, the structures of single catalytic centers of Ni(OH)_2_-type NPs embedded in SaltMe and *meso*-SaldMe matrices were optimized with the use of quantum chemical calculations on the DFT level (Figure S12). Both optimized structures indicated very high stability manifested by the negative values of the sum of electronic and thermal free energies. The nickel center of Ni(OH)_2_ embedded in *meso*-SaldMe is positioned in front of the space-folded plane of the *meso*-SaldMe matrix, while the nickel center of Ni(OH)_2_ embedded in SaltMe is placed in the matrix plane and stabilized in this position by hydrogen bonding to imine nitrogen. The axial coordination of ethanol was easy because of facilitated access of ethanol molecules to active centers of the Ni(OH)_2_-type NP catalyst derived from poly(*meso*-NiSaldMe)-PS (Figure S12).

Methodologies for devising Ni-based electrocatalysts are limited. Ni oxides and Ni hydroxides are often prepared by long hydro- and solvothermal processes followed by high-temperature treatment methods. These processes are accompanied by heavy energy consumption. The approach presented here is facile, energy-saving, and reproducible for electrocatalysts with excellent performance. The current densities obtained from both Ni(OH)_2_-type NPs are better than similar reported materials (Table 4). To conclude, poly(Salen) films performed much better than salen complexes immobilized by zeolite over the electrode surface.

**Table 4:** Comparison of the Electrocatalytic Activity of Ni Salen-Based Materials in Ethanol Electrocatalysis^a^

substrate	*C*_NaOH_ (M)	*C*_EtOH_ (M)	current density (maximal) (mA cm^–2^)	scan rate (mV s^–1^)	refs
poly[Ni(Salen)]/GCE	1.0	0.1	16.5	50	(38)
poly[Ni(3-MeOSalen)]-GCE	0.2	1.0	10	50	(37)
poly[Ni(α,α′-methyl_2_salen)]/GCE	0.1	0.3	15	50	(61)
poly[Ni(II)-DHS]/GCE	0.1	0.1	2.5	20	(64)
poly[VP-*co*-DVB-Ni(II)]/CE	0.5	1.0	∼0.35	15	(65)
Ni^II^{salnptn(4-OH)_2_}-zeolite-Y/CE	1.0	0.49	6.2	20	(66)
poly[Ni^II^{salnptn(4-OH)_2_}]/Pt	1.0	0.12	∼23.5	20	(66)
poly[*meso*-SaldMe]-PS_low_/CPE	0.2	0.3	29.6	50	this work

a GCE—glassy carbon electrode, CE—carbon paste electrode, CPE—carbon paper electrode, VP-*co*-DVB—4-vinylpyridine-co-divinylbenzene.

### Effect of Addition of RGO on Catalytic Performance

The remarkable performances of the carbon materials incorporated with polymer films were widely studied.^67−69^ The conjugated electronic structure, intrinsic conductivity, high surface area, and mechanical strength reflected in reinforcing properties of RGO were found to be responsible for improved properties of poly(NiSalen) films.^40^

The poly(NiSaltMe) and poly(*meso*-NiSaldMe) films were deposited at a high scan rate on the CPE drop-coated with RGO (Figure S13a,b). The electropolymerization range was extended to 1.4 V in PD deposition. As expected, currents for both monomers’ oxidation were high (Figure S13) in comparison to oxidation currents obtained over bare CPE (Figures 1 and 2). The other observed change during the multi-cyclic PD electropolymerization of NiSaltMe and poly(*meso*-NiSaldMe) on the RGO-reinforced CPEs was the oxidation and reduction peak broadening (Figure S13). The charges passed during RGO-poly(NiSaltMe)-PD_high_ and RGO-poly(*meso*-NiSaldMe)-PD_high_ depositions were 578 and 621 mC cm^–2^, respectively. The Ni(OH)_2_ NP generation step confirmed a very stable composite system (Figure S14). Expectedly, NP generation took much more time (Figure S14). Therefore, to obtain a stable current of oxidation of Ni^2+^/Ni^3+^ and reduction of Ni^3+^/Ni^2+^ peaks, at least 150–300 CV cycles in 0.2 M NaOH_aq_ were required. Also, the potential range during NP generation was enlarged to 1.5 V.

Figures S15 and S16 show cyclic voltammograms in 0.2 M NaOH_aq_ at different ethanol concentrations on Ni(OH)_2_-type NPs derived from RGO-poly(NiSaltMe)-PD_high_ and RGO-poly(*meso*-NiSaldMe)-PD_high_ films. The lowest catalytic performances were obtained for poly(NiSaltMe) potentiodynamically deposited with a high scan rate (Figure S15). The NP catalytic efficiency was lower when polymers were deposited on the drop-coated RGO CPE. This performance was worse than that of the NPs derived from poly(NiSalen)s deposited over bare CPE (Table 1). We speculate that because of the deposition of a highly stable film in the presence of RGO, only part of the polymers generated NPs. This low population of NPs resulted in a low catalytic efficiency.

## Conclusions

Two nickel salen polymers, poly(*meso*-NiSaldMe) and poly(NiSaltMe), were deposited on CPEs under PD_low_, PD_low′,_ PD_high_, PD_high′,_ PS_low,_ and PS_high_ conditions. These different polymerization conditions influenced polymer morphology. Under basic conditions, the primary target of electron release changed from the salen ligand center to the nickel center, which led to the synthesis of Ni(OH)_2_ NPs embedded in the poly(Salen) matrix. The morphology of poly(NiSalen) films influenced the arrangement of NPs in poly(Salen) matrixes. A compact homogeneous poly(NiSaltMe) film arranged NPs in a 2D matrix, whereas a spatially extended globular poly(*meso*-NiSaldMe) film arranged NPs in a 3D matrix. A globular poly(*meso*-NiSaldMe) precursor required more CV cycles to complete transformations into NPs than poly(NiSaltMe). XPS analysis confirmed the loss of square-planar geometry of Ni^2+^ after polymer transformation into NPs, and DFT calculations optimized the geometry of Ni(OH)_2_ active centers embedded in the poly(Salen) matrix.

Estimated *A*_ECSA_ values were 17.5 and 33.0 cm^2^ for NPs derived from poly(NiSaltMe)-PS_high_ and poly(*meso*-NiSaldMe)-P_Slow_, respectively. NPs derived from poly(*meso*-NiSaldMe) revealed a higher catalytic performance than NPs derived from poly(NiSaltMe). Ni(OH)_2_ NPs derived from both polymers demonstrate catalytic performance toward ethanol in the concentration range of 0.05 to 0.5 M. In this electrocatalysis process, the charge transfer was the controlling step, and the redox equilibrium did not prevail. The main product of electro-oxidation of ethanol was recognized as acetic acid.

EIS results indicated that the charge-transfer processes, that is, heterogeneous redox Ni^2+^/Ni^3+^reactions occurring at the interface of CPE|NPs surrounded by the poly(Salen) matrix, were fast. However, charge-transfer processes were slower during electrocatalytic oxidation of ethanol. The catalytic charge-transfer process was faster for the NP catalyst derived from poly(*meso*-NiSaldMe)-PS_low_ than for the NP catalyst derived from poly(NiSaltMe)-PS_high_. Furthermore, NPs can maintain their catalytic activity for up to 1 month, with the possibility of multiple regenerations. These electrogenerated NPs were significantly resistant to poisoning effects and, therefore, can be used multiple times.

## Supporting Information Available

PS depositions of poly(NiSaltMe)-PS_low_ and poly(NiSaltMe)-PS_high_; multi-cyclic curves of potential-driven NP generation from poly(NiSaltMe)-PS_low_ and poly(NiSaltMe)-PS_high_; PS depositions of poly(*meso*-NiSaldMe)-PS_low_ and poly(*meso*-NiSaldMe)-PS_high_; multi-cyclic curves of potential-driven NP generation from poly(*meso*-NiSaldMe)-PS_low_ and poly(*meso*-NiSaldMe)-PS_high_; SEM images of a bare carbon paper; Ni XPS spectra of poly(*meso*-NiSaldMe)s and poly(NiSaltMe)s and NPs generated from them; catalytic stability; SEM and XPS measurements after ethanol electro-oxidation; determination of equilibration time for EIS measurements; cyclic voltammograms of Ni(OH)_2_-type NPs derived from poly(NiSaltMe)-PS_high_ and poly(*meso*-NiSaldMe)-PS_low_ in 0.1 M NaOH and 0.08 M C_2_O_4_^2–^; DFT level-optimized molecular structures of single catalytic centers of NPs of Ni(OH)_2_ embedded in the SaltMe and *meso*-SaldMe matrix; electropolymerization of NiSaltMe and *meso*-NiSaldMe over the RGO-coated CPE electrode; NP generation and catalytic CV responses of Ni(OH)_2_-type NPs derived from CPE/RGO-poly(*meso*-NiSaldMe)-PD_high_ and CPE/RGO-poly(NiSaltMe)-PD_high_; summary of peak potentials and peak current values for Ni(OH)_2_-type NPs derived from poly(NiSaltMe)-PS_high_ and poly(*meso*-NiSaldMe)-PS_low_ in NaOH and in the presence of ethanol; values of the circuit elements obtained by fitting EIS experimental data performed at potentials involving heterogeneous redox Ni^2+^/Ni^3+^ reactions for both Ni(OH)_2_-type NPs in the presence and absence of ethanol; and electrochemically active surface area (*A*_ECSA_) estimated by the oxalate method (PDF)
