# Supplementary material for: Ni(OH)2-Type Nanoparticles Derived from Ni Salen Polymers: Structural Design toward Functional Materials for Improved Electrocatalytic Performance
Source: ACS Appl Mater Interfaces. 2022 Jul 15;14(29):33768–86. doi: 10.1021/acsami.2c06147 (PMC9335556; doi:10.1021/acsami.2c06147)
Supplement: Supplementary file 1 — am2c06147_si_001.pdf [file am2c06147_si_001.pdf]

Supplementary Information

$Ni(OH)_2$ type nanoparticles derived from Ni salen polymers: Structural design toward functional material for improved electrocatalytic performance

Monika Mierzejewska, Kamila Łępicka, Jakub Kalecki, Wojciech Lisowski,*

*and Piyush Sindhu Sharma**

Institute of Physical Chemistry, Polish Academy of Sciences,

Kasprzaka 44/52, 01-224 Warsaw, Poland

Corresponding Authors

*E-mails: psharma@ichf.edu.pl (Piyush Sindhu Sharma),

klepicka@ichf.edu.pl (Kamila Łępicka)

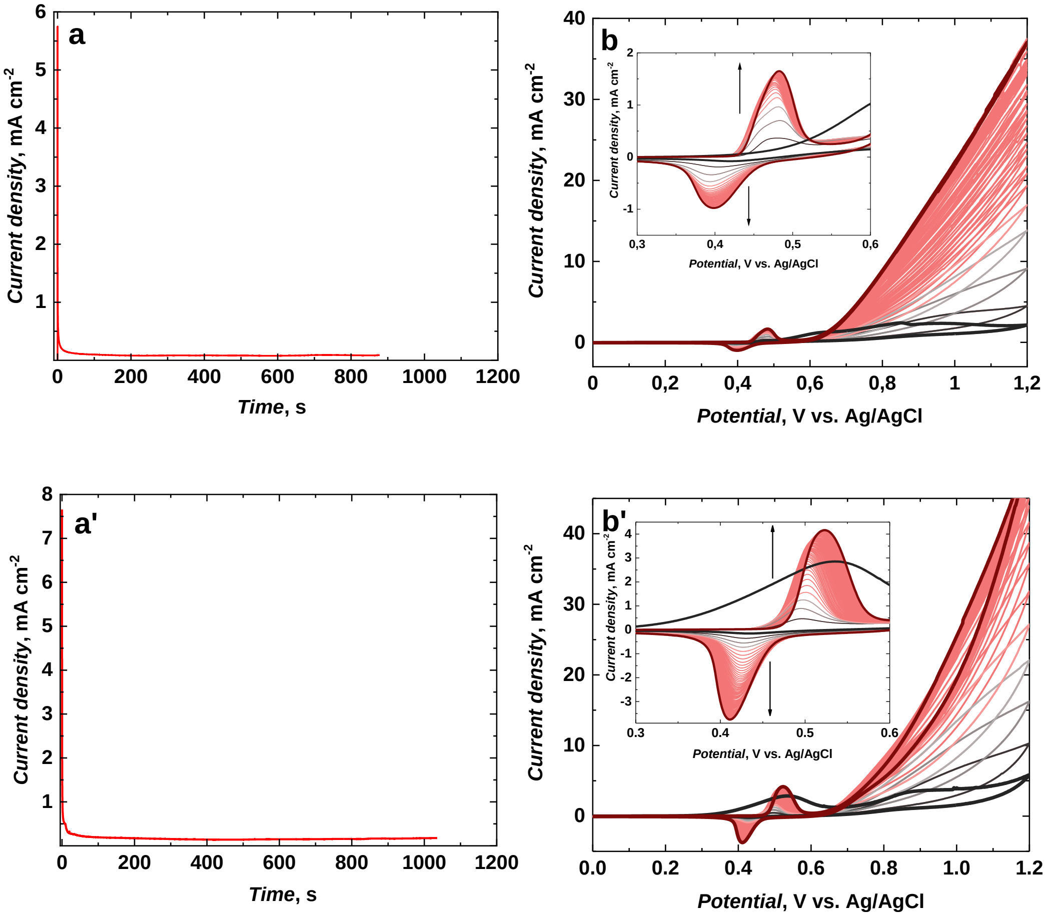

Figure S1. Potentiostatic depositions of (a) poly(NiSaltMe)-PS_{low} and (a') poly(NiSaltMe)-PS_{high}. Multi-cyclic curves of potential driven NPs generation from (b) poly(NiSaltMe)-PS_{low} and (b') poly(NiSaltMe)-PS_{high} performed in 0.2 M $NaOH_{aq}$ at 20 mV s^{-1}.

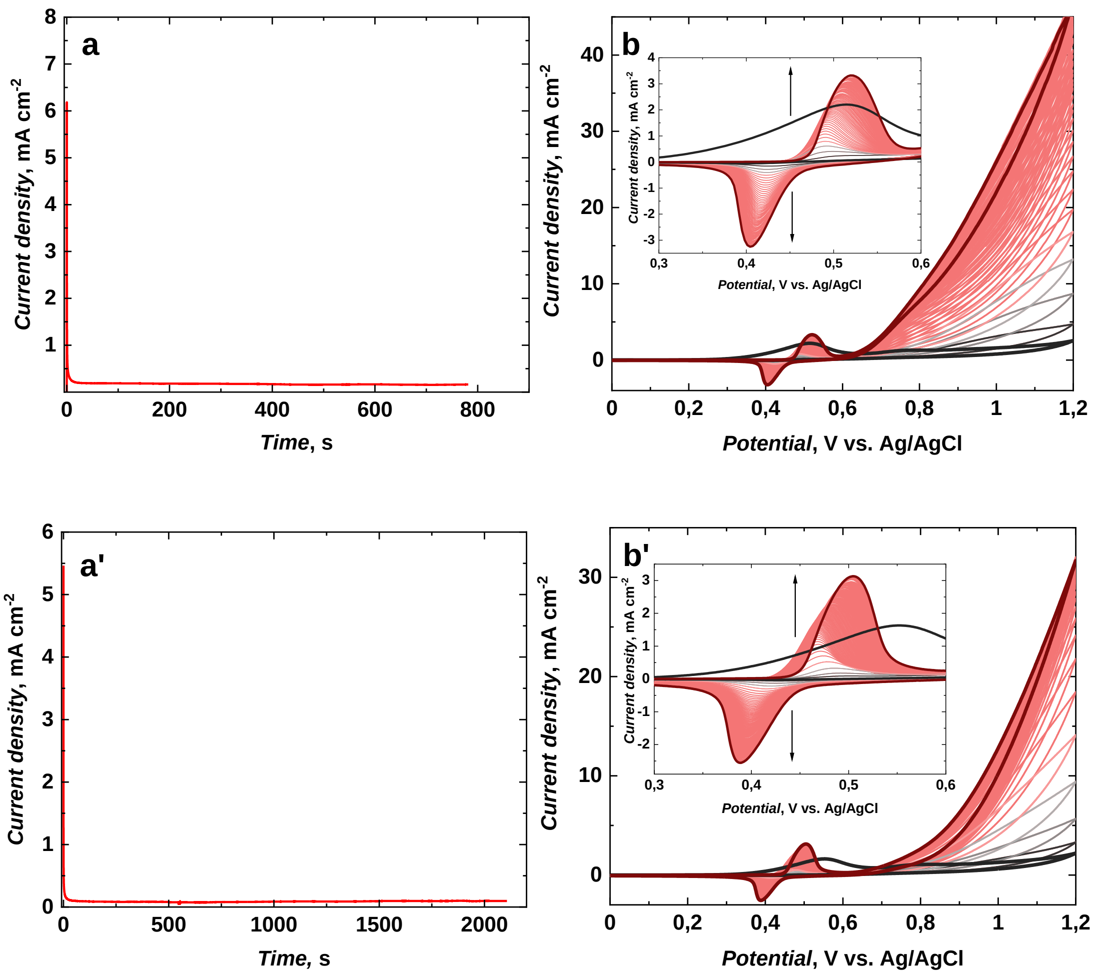

Figure S2. Potentiostatic depositions of (a) poly(*meso*-NiSaldMe)-PS_{low} and (a’) poly(*meso*-NiSaldMe)-PS_{high}. Multi-cyclic curves of potential driven NPs generation from (b) poly(*meso*-NiSaldMe)-PS_{low} and (b’) poly(*meso*-NiSaldMe)-PS_{high} performed in 0.2 M $NaOH_{aq}$ at 20 mV s^{-1}.

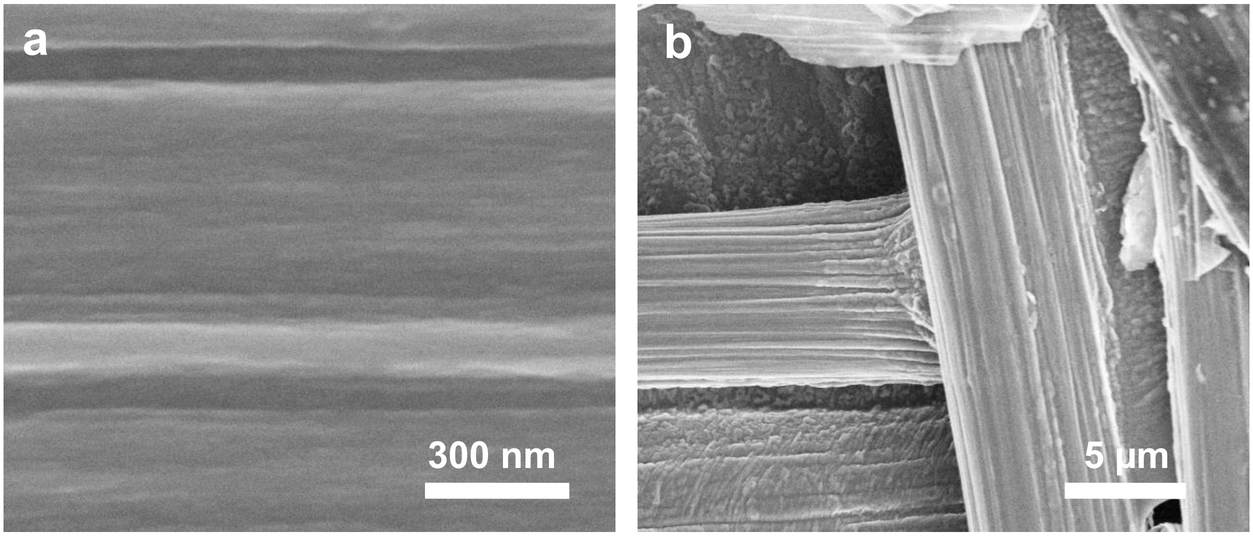

Figure S3. SEM images of a bare carbon paper at the two different magnifications.

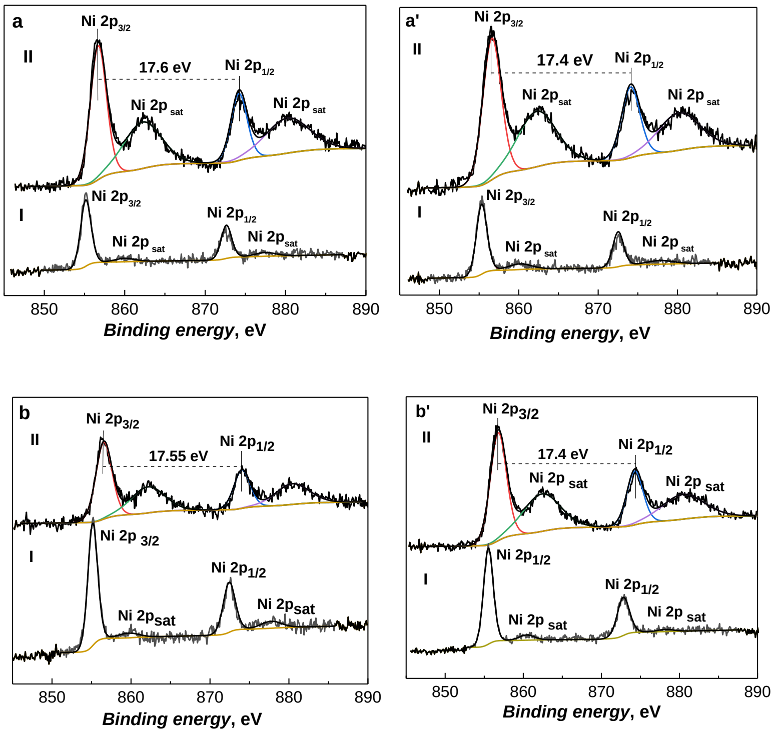

Figure S4. Ni XPS spectra of (a, and a') (I) poly(*meso*-NiSaldMe) and (II) NPs derived from poly(*meso*-NiSaldMe), (b, and b') (I) poly(NiSaltMe) and (II) NPs generated from poly(NiSaltMe). Poly(meso-NiSaldMe): a – PD_{high} a' – PD_{low}; poly(NiSaltMe): b – PD_{high} b' – PD_{low}

The O 1s XPS spectra for poly(NiSaltMe) (Fig S5a) and poly(*meso*-NiSaldMe) (Fig S5b) showed a peak at 530.2 eV corresponding to C-**O**-Ni.[1] Figure S5a(II) and b(II) showed O 1s XPS spectra for $Ni(OH)_2$ type NPs derived from poly(NiSaltMe) (Fig. S5a) and poly(*meso*-NiSaldMe) (Fig. S5b). These spectra were deconvoluted into two peaks. Peaks appearing at 532.1 and 533.3 eV corresponded to Ni-**O**, and **O**H^- from $Ni(OH)_2$, respectively.[2] More importantly, it indicated that NPs generation step resulted in oxygenated Ni, as shown in the inset scheme in Figure S5.

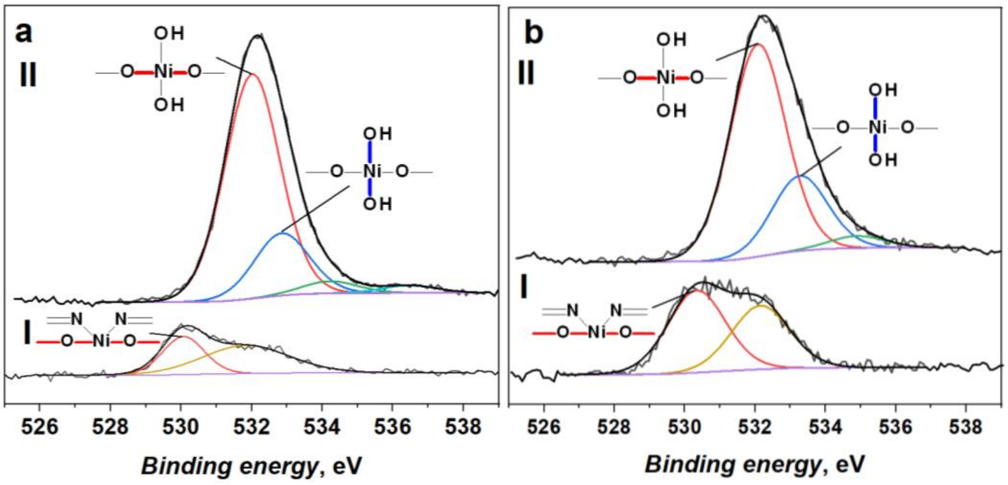

Figure S5. O 1s XPS spectra of (a I) poly(NiSaltMe)-PS_{low} and (a II) NPs derived from poly(NiSaltMe)-PS_{low}, (b I) poly(*meso*-NiSaldMe)-PS_{high} and (b II) NPs generated from poly(*meso*-NiSaldMe)-PS_{high}.

Similarly, summarizations of C 1s XPS spectra for poly(NiSaltMe) (Fig S6a) and poly(*meso*-NiSaldMe) (Fig S6b) are shown below.

Poly(NiSaltMe)

C sp^2 (283.7 eV), C sp^3 (284.8 eV), C=N (285.3 eV) and C_{Ar}-O (286.5 eV)

NPs generated from poly(NiSaltMe)

C sp^2 (283.7 eV), C sp^3 (284.8 eV), C=N (285.3 eV) and C_{Ar}-O (286.1 eV)

Poly(*meso*-NiSaldMe)

C sp^2 (283.5 eV), C sp^3 (284.6 eV), C=N (285.5 eV) and C_{Ar}-O (286.6 eV)

NPs generated Poly(*meso*-NiSaldMe)

C sp^2 (284.2 eV), C sp^3 (284.8 eV), C=N (285.3 eV) and C_{Ar}-O (286 eV)

No change in C 1s peaks positioning indicates that states of Salen ligands' carbon atoms were in the same state in the poly(Salen) films and NPs in poly(Salen) matrix samples.

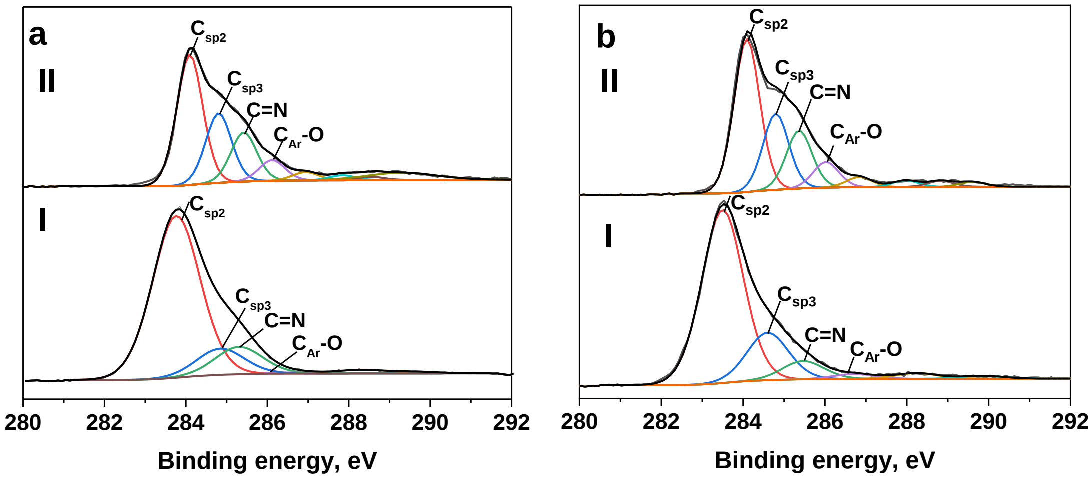

Figure S6. C 1s XPS spectra of (a I) poly(NiSaltMe)-PS_{low} and (a II) NPs derived from poly(NiSaltMe), (b I) poly(*meso*-NiSaldMe)-PS_{high} and (b II) NPs generated from poly(*meso*-NiSaldMe).

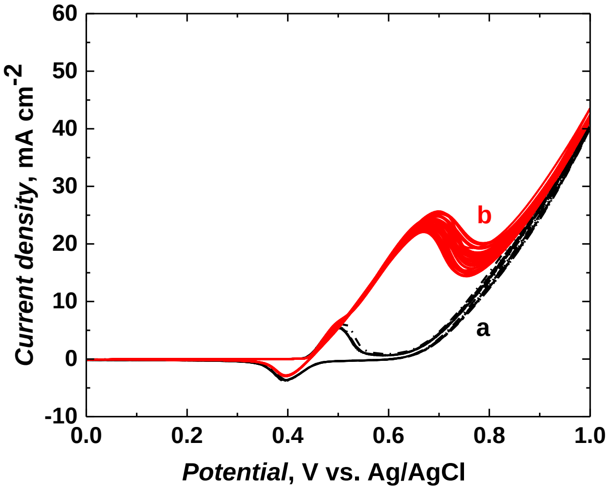

Figure S7. Extensive CV cycling of $Ni(OH)_2$ NPs generated from the poly(NiSaltMe)-PS_{high} in 0.2 M $NaOH_{aq}$ at 50 mV s^{-1}, in the (a) absence and (b) presence of 0.3 M ethanol.

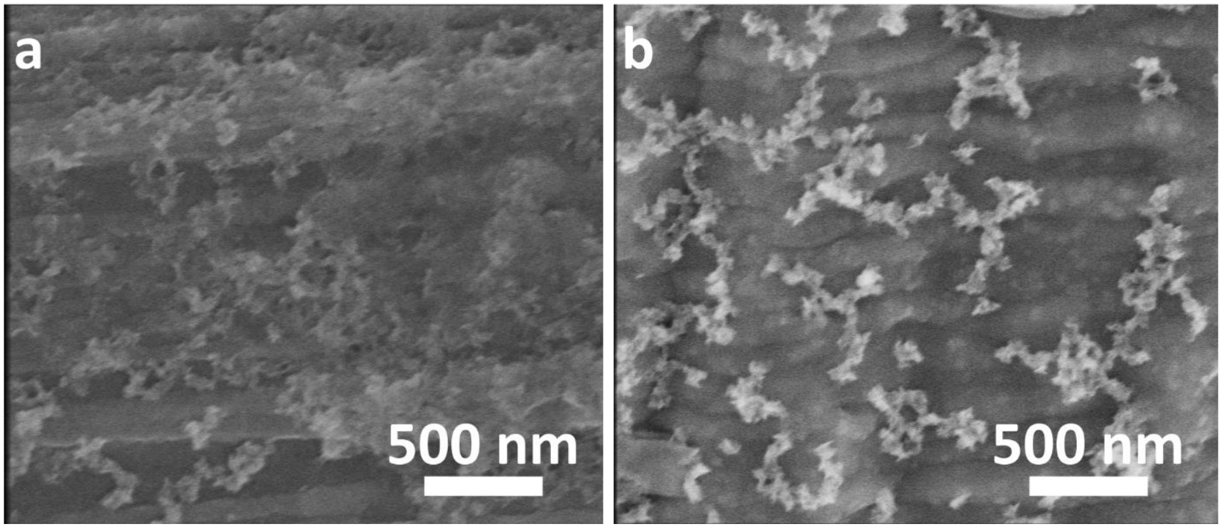

Figure S8. SEM images of $Ni(OH)_2$ type nanoparticles derived from (a) poly(NiSaltMe)-PS_{high}, (b) poly(*meso*-NiSaldMe)-PS_{low} after ethanol electrooxidation.

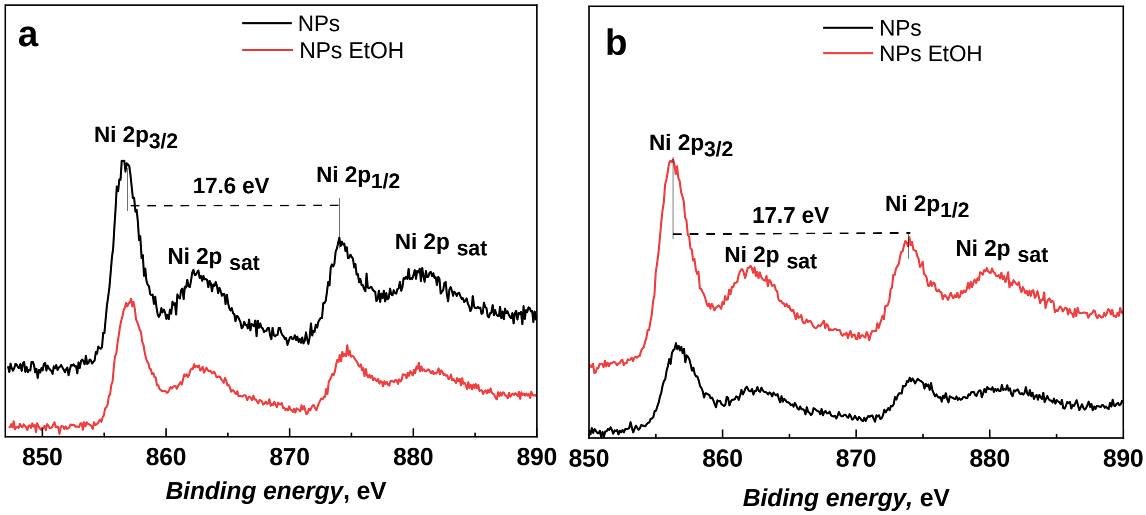

Figure S9. Ni 2p XPS spectra, before (black) and after (red) catalytic electrooxidation of ethanol on NPs generated from (a) poly(NiSaltMe)-PS_{high}, and (b) poly(*meso*-NiSaldMe)-PS_{low}.

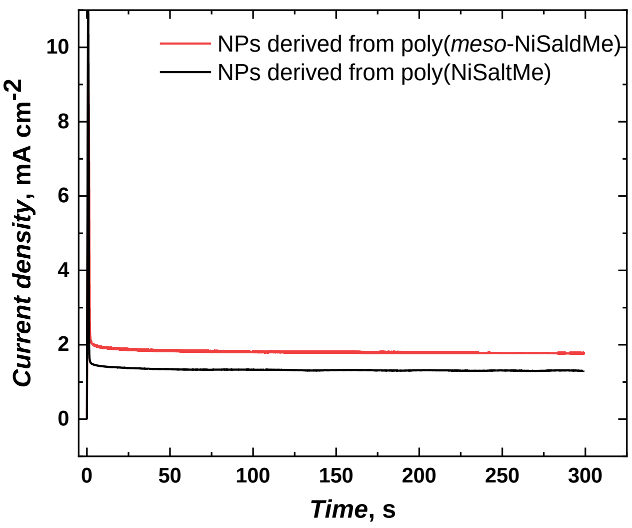

Figure S10. Chronoamperometric determination of time needed to reach current equilibrium for the $Ni(OH)_2$ type NPs derived from poly(NiSaltMe)-PS_{high} (black) and poly(*meso*-NiSaldMe)-PS_{low} (red) at 0.6 V in 0.2 M $NaOH_{aq}$.

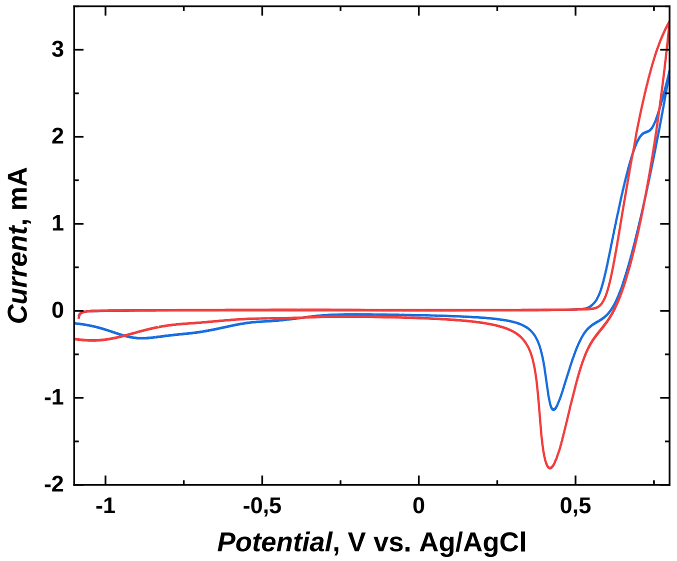

Figure S11. Cyclic voltammograms of $Ni(OH)_2$ type NPs derived from poly(NiSaltMe)-PS_{high} (blue) and poly(*meso*-NiSaldMe)-PS_{low} (red) at 150 mV s^{-1} in 0.1 M NaOH and 0.08 M $C_2O_4^{2-}$.

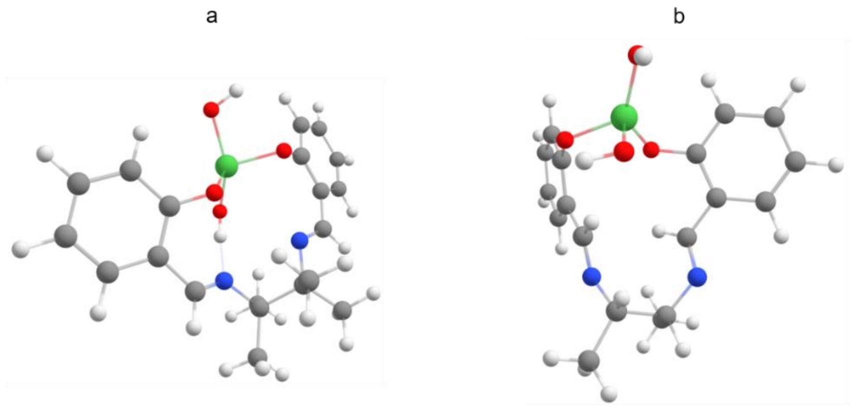

Sum of Electronic and thermal Free Energies of the Optimized [Ni(OH)$_2$SaltMe] NP unit -7076045.0 kJ mol^{-1}

Sum of Electronic and thermal Free Energies of the Optimized [Ni(OH)$_2$meso-SaldMe] NP unit −6869703.7 kJ mol^{-1}

Stability of Ni-(OH)$_2$ *meso*-SaldMe

Complex	Calculated quantity	Energy of complex [kJ/mol]	Sum Energies of components [kJ/mol]	Binding Energies of complex [kJ/mol]
**Ni-(OH)$_2$ *meso*-SaldMe**	Sum of Electronic and Zero-Point Energies	−6869559.9	−6869457.9	−102.0
**Ni-(OH)$_2$ *meso*-SaldMe**	Sum of Electronic and thermal Free Energies	−6869703.7	−6869671.6	−32.1

Stability of Ni-(OH)$_2$SaltMe

Complex	Calculated quantity	Energy of complex [kJ/mol]	Sum Energies of components [kJ/mol]	Binding Energies of complex [kJ/mol]
**Ni-(OH)$_2$SaltMe**	Sum of Electronic and Zero-Point Energies	−7075890.0	−7075820.5	−79.5
**Ni-(OH)$_2$SaltMe**	Sum of Electronic and thermal Free Energies	−7076045.0	−7076040.0	−5.0

Figure S12. The DFT level optimized molecular structures of single catalytic centers of NPs of Ni(OH)$_2$ embedded in (a) SaltMe and (b) *meso*-SaldMe matrix with the use of Tao-Perdew Staroverov-Scuseria (TPSS) functional. The values of the binding energies of complex in both cases represented catalytic unit stability.

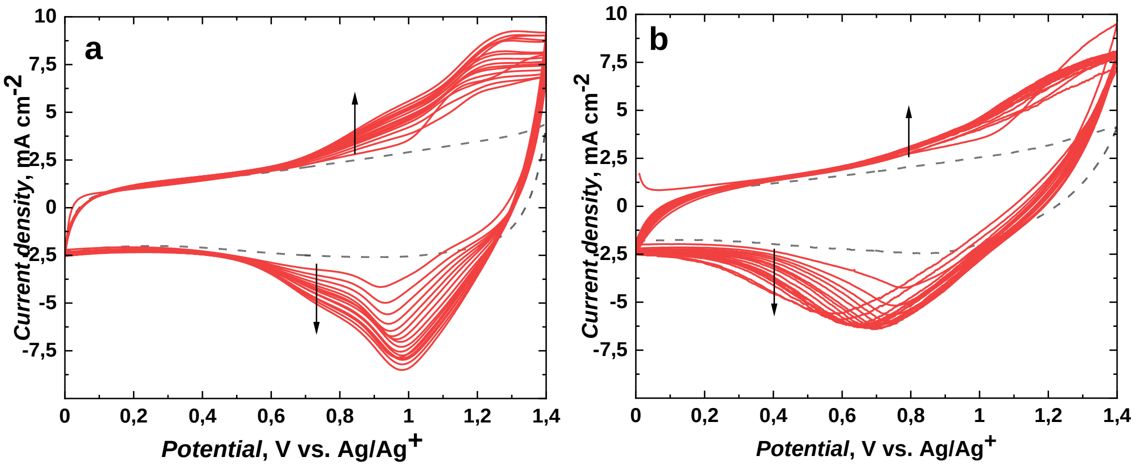

Figure S13. Multi-cyclic potentiodynamic curves (red) of oxidative electropolymerization of 1 mM (a) NiSaltMe, and (b) *meso*-NiSaldMe at 100 mV s^{-1} in an acetonitrile solution of 0.1 M $(TBA)PF_6$. Black curve (dash) represents CV performed over the RGO coated CPE electrode without monomers in an acetonitrile solution of 0.1 M $(TBA)PF_6$.

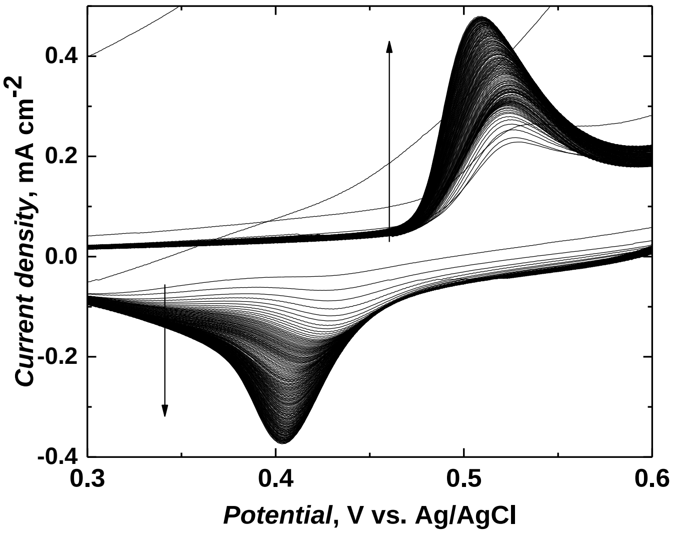

Figure S14. Multi-cyclic curves of potential driven NPs generation from CP/RGO-poly(NiSaltMe)-PD_{high} at 20 mV s^{-1} in 0.2 M $NaOH_{aq}$.

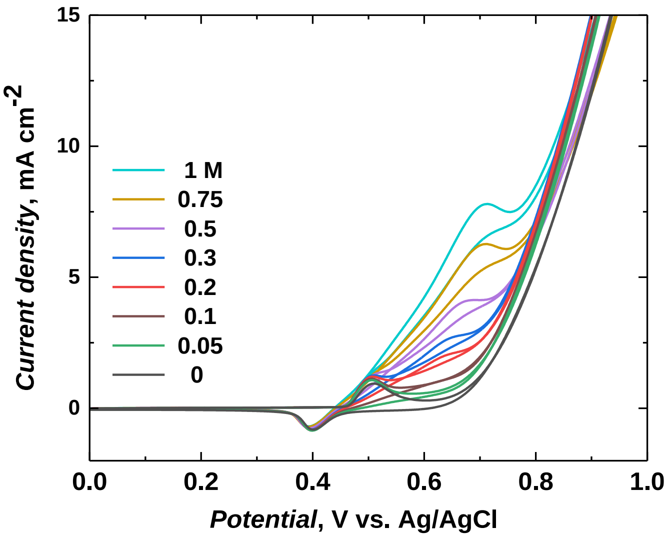

Figure S15. The catalytic CV responses of $Ni(OH)_2$ type NPs derived from CPE/RGO-poly(NiSaltMe)-PD_{high}, towards various ethanol concentrations performed at 50 mV s^{-1} in 0.2 M $NaOH_{aq}$.

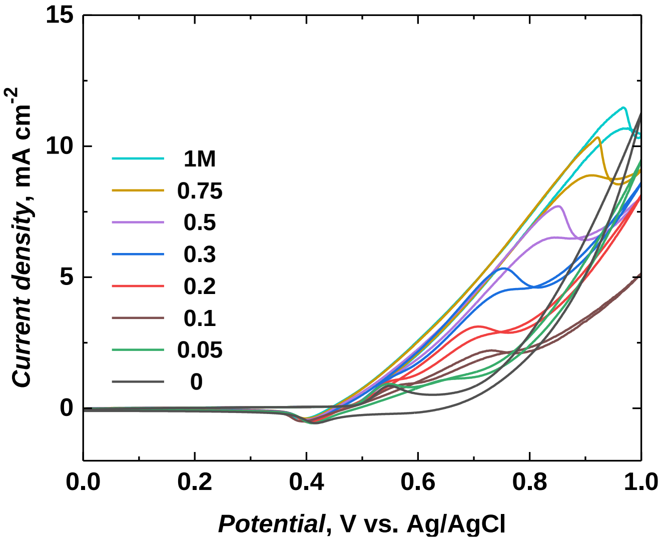

Figure S16. The catalytic CV responses of $Ni(OH)_2$ type NPs derived from CP/RGO- Poly(*meso*-NiSaldMe)-PD_{high}, towards various ethanol concentrations performed at 50 mV s^{-1} in 0.2 M $NaOH_{aq}$.

Table S1. Peak potentials and peak currents values determined from the multi-scan rate experiment performed for $Ni(OH)_2$ type NPs derived from poly(NiSaltMe)-PS_{high} in 0.2 M $NaOH_{aq}$.

**v**<br>mV s^{-1}	I_{pa}	I_{pc}<br>mA	E_{pa}	E_{pc}<br>V vs. Ag/AgCl	ΔE
2	0.108	-0.091	0.493	0.429	0.064
5	0.248	-0.200	0.500	0.424	0.076
10	0.430	-0.368	0.508	0.419	0.089
30	0.977	-0.837	0.528	0.406	0.122
50	1.377	-1.197	0.540	0.395	0.145
100	2.134	-1.848	0.562	0.379	0.183
200	3.194	-2.821	0.594	0.356	0.238

Table S2. Peak potentials and peak currents values determined from the multi-scan rate experiment performed for $Ni(OH)_2$ type NPs derived from poly(*meso*-NiSaldMe)-PS_{low} in 0.2 M $NaOH_{aq}$.

v<br>mV s^{-1}	I_{pa}	I_{pc}<br>mA	E_{pa}	E_{pc}<br>V vs. Ag/AgCl	ΔE
2	0.146	-0.120	0.501	0.432	0.069
5	0.313	-0.272	0.509	0.426	0.083
10	0.543	-0.48	0.517	0.420	0.097
30	1.113	-1.019	0.537	0.404	0.133
50	1.509	-1.378	0.551	0.39	0.161
100	2.220	-1.977	0.575	0.369	0.206
200	3.256	-2.622	0.601	0.337	0.264

Table S3. Peak potentials and peak currents values determined from the multi-scan rate experiment performed for $Ni(OH)_2$ type NPs derived from poly(NiSaltMe)-PS_{high} and poly(*meso*-NiSaldMe)-PS_{low} in 0.2 M $NaOH_{aq}$ in the presence of 0.3 M ethanol.

	NPs derived from poly(NiSaltMe)-PS_{high}					NPs derived from poly(*meso*-NiSaldMe)-PS_{low}				
v	E_a	E_c	ΔE	I_a	I_c	E_a	E_c	ΔE	I_a	I_c
V s^{-1}	V vs Ag/AgCl			mA		V vs Ag/AgCl			mA	
0.002	0.678	0.416	0.262	4.082	-0.014	0.658	0.424	0.234	2.857	-0.029
0.005	0.675	0.424	0.251	4.131	-0.063	0.665	0.427	0.238	3.161	-0.079
0.01	0.676	0.423	0.253	4.191	-0.144	0.673	0.425	0.248	3.370	-0.186
0.03	0.678	0.414	0.264	4.464	-0.459	0.676	0.414	0.262	3.641	-0.522
0.05	0.685	0.408	0.277	4.580	-0.681	0.683	0.406	0.277	3.751	-0.789
0.1	0.689	0.395	0.294	4.783	-1.196	0.687	0.391	0.296	3.982	-1.295
0.2	0.694	0.376	0.318	5.099	-1.914	0.697	0.367	0.330	4.435	-2.044

Table S4. Values of the circuit elements obtained by fitting EIS experimental data performed at potentials involving heterogeneous redox Ni^{2+}/Ni^{3+}reactions (Fig.12 and 14) to the equivalent circuit (Fig.12c and Fig.14c) for both $Ni(OH)_2$ type NPs in the presence and absence of ethanol.

		**NPs derived from poly(NiSaltMe)**				**NPs derived from poly(*meso*-NiSaldMe)**			
Concentration (M)	Potential (V)	R_Ω (Ω)	R_{ct} (Ω)	C_{dl} (mF)	p	R_Ω (Ω)	R_{ct} (Ω)	C_{dl} (mF)	p
0	0.60	44.27	61.67	0.48	0.9469	44.60	46.83	0.76	0.9354
0	0.65	44.86	21.19	0.45	0.9146	44.44	17.29	0.69	0.9142
0.3	0.65	48.22	52.87	0.45	0.9621	45.68	31.27	0.68	0.9747

Double layer capacitance (C_{dl}) was calculated for constant phase element (*CPE'*) corresponding to the non-ideal capacitor character of the studied material.[3] Coefficient p defines *CPE'*. Value of p varies from 0 to 1, corresponding to a pure resistor and ideal capacitor, respectively. C_{dl} was calculated from *CPE'* according to equation (S1):

$$C_{dl} = CPE' \cdot (\omega")^{p-1} \quad \text{(S1)}$$

Where ω" is the maximum frequency of the imaginary part of the impedance.

Electrochemically active surface area (A_{ECSA}) was estimated with the oxalate method. For both $Ni(OH)_2$ type NPs derived from poly(NiSaltMe)-PS_{high} and poly(*meso*-NiSaldMe)-PS_{low} have registered CV curves in 0.1 M NaOH and 0.08 M $C_2O_4^{2-}$ with the scan rate 150 mV s^{-1}. Such a high scan rate ensures stabilization of NPs surface and limits growth to a monolayer. Herein surface hydroxide groups are substituted with oxalate ions and form five-member rings. The charge (Q) was estimated from the discharging part of a CV curve in potential window 0.25- 0.58 V, with the relation I= f(t), was estimated charge (Q). Hall et al.[4] proposed a method using a theoretical value of a specific charge (q) of 195 μC cm^{-2}. This value was determined with the unit cell parameters for hexagonal α-$Ni(OH)_2$. For β-$Ni(OH)_2$ value of the specific charge is 198.2 μC cm^{-2} due to different Ni-Ni spacing (3.08 Å and 3.13 Å for α-$Ni(OH)_2$ and β-$Ni(OH)_2$, respectively). Including geometrical area of the electrode used in the experiment, specific charge employed to measure active surface area was 38.8 μC cm^{-2}. Finally, A_{ECSA} were determined with equation (S2)

$$A_{ECSA}[cm^2] = \frac{Q\ [mC]}{q[\frac{mC}{cm^2}]}, \quad \text{(S2)}$$

$$\text{where as } Q = \int_{V_1}^{V_2} I(t)\ dV \quad \text{(S3)}$$

Estimated A_{ECSA} with this method was 17.5 $cm^{2,}$ and 33.0 cm^2 for NPs derived from poly(NiSaltMe)-PS_{high} and poly(*meso*-NiSaldMe)-PS_{low}, respectively. These values support results for surface concentrations of Ni^{3+} oxy-hydroxide, where $Ni(OH)_2$ type NPs derived from poly(*meso*-NiSaldMe)-PS_{low} indicate a higher amount of active redox species.

Reference

(1) Deng, F.; Li, X.; Ding, F.; Niu, B.; Li, J. Pseudocapacitive Energy Storage in Schiff Base Polymer with Salphen-Type Ligands, *J. Phys. Chem*. C **2018**, 122, 5325-5333.

(2) Karikalan, N.; Velmurugan, M.; Chen, S.-M.; Karuppiah, C. Modern Approach to the Synthesis of $Ni(OH)_2$ Decorated Sulfur Doped Carbon Nanoparticles for the Nonenzymatic Glucose Sensor. *ACS Appl. Mater. Interfaces* **2016,** 8, 22545-22553.

(3) C. H. Hsu, F. Mansfeld, Technical Note: Concerning the Conversion of the Constant Phase Element Parameter Y_0 into a Capacitance, *Corrosion*, **2001**, *57*, 747- 748.

(4) Hall, D. S.; Bock, C.; MacDougalla, B. R. An Oxalate Method for Measuring the Surface Area of Nickel Electrodes. *J. Electrochem. Soc.* **2014,** *161*, H787-H795.
